# Förster Resonance Energy Transfer between Quantum Dot Donors and Quantum Dot Acceptors

**DOI:** 10.3390/s150613288

**Published:** 2015-06-05

**Authors:** Kenny F. Chou, Allison M. Dennis

**Affiliations:** 1 Department of Biomedical Engineering, Boston University, Boston, MA 02215, USA; E-Mail: kfchou@bu.edu; 2 Department of Biomedical Engineering and Division of Materials Science and Engineering, Boston University, Boston, MA 02215, USA

**Keywords:** Förster resonance energy transfer, FRET, semiconductor quantum dots, biosensing, resonance energy transfer, crosstalk, solar harvesting

## Abstract

Förster (or fluorescence) resonance energy transfer amongst semiconductor quantum dots (QDs) is reviewed, with particular interest in biosensing applications. The unique optical properties of QDs provide certain advantages and also specific challenges with regards to sensor design, compared to other FRET systems. The brightness and photostability of QDs make them attractive for highly sensitive sensing and long-term, repetitive imaging applications, respectively, but the overlapping donor and acceptor excitation signals that arise when QDs serve as both the donor and acceptor lead to high background signals from direct excitation of the acceptor. The fundamentals of FRET within a nominally homogeneous QD population as well as energy transfer between two distinct colors of QDs are discussed. Examples of successful sensors are highlighted, as is cascading FRET, which can be used for solar harvesting.

## 1. Introduction

Förster (or fluorescence) resonance energy transfer (FRET) is a non-radiative energy transfer process from a fluorescent donor to a lower energy acceptor via long-range dipole-dipole interactions [1,2]. FRET-based technologies have played a significant role in biosensing, where FRET is used as a “molecular ruler” to measure and image changes in biomolecular conformations, nucleic acid hybridization, enzyme activity, and environmental parameters, such as pH [3,4,5,6,7]. FRET has also garnered interest for its potential in other applications, such as photonic logic gates [8,9] and solar energy harvesting applications [10,11,12,13].

Any fluorescent moiety, including organic small-molecule dyes, fluorescent proteins (FPs), lanthanide dyes, and fluorescent nanoparticles (NPs), can be used as a FRET donor [14]. In particular, semiconductor nanocrystal (NC) quantum dots (QDs) exhibit excellent photophysical properties that are highly desired in a FRET donor, including: (1) broad absorption spectra; (2) large absorption cross-sections; (3) narrow, size-tunable emission spectra; (4) long fluorescence lifetimes; (5) bright and photostable emission; and (6) a large effective Stokes shift [5,15,16,17,18,19,20,21]. In addition to the advantageous photophysical characteristics of QDs, their nanoparticle structure also presents a large, biochemically-accessible surface area, facilitating the incorporation of multiple biomolecules or dyes into a single QD-centered biosensing device [5,22]. The benefits of using QDs as a FRET donor in hybrid systems are well documented and multiple in-depth reviews have been published on the topic [3,4,23,24,25,26,27].

Two types of acceptor molecules can be employed in FRET: quenchers (non-fluorescent energy acceptors) or fluorophores [2,14]. Metal nanoparticles and organic molecules can play the role of a quencher, while fluorescent acceptors are typically organic dyes or fluorescent proteins [2,14]. For QDs to be effective fluorescent acceptors in hybrid systems, their long fluorescence lifetime necessitates that the donor moiety also emit with a comparably long radiative lifetime for measurable FRET to be observed [28]. For this reason, QDs have been more effective as acceptors in systems with long-lived lanthanide dyes as the donor [29,30] or in related energy transfer processes like bioluminescence resonance energy transfer (BRET) [31,32,33] and chemiluminescence resonance energy transfer (CRET) [34], where the donor excitation is initiated by a chemical reaction rather than external illumination. QDs exhibit negligible FRET with traditional organic dyes as the donor [28].

Although QDs exhibit excellent spectral properties, small organic dyes and fluorescent proteins are still preferred as the donor and/or acceptor molecules in many sensing applications. As Resch-Genger *et al.* suggest in their review, QDs should only be used if the specific properties imparted by their unique photophysical characteristics are highly desired in the application at hand [15]. This caution applies doubly in the case of QD-QD FRET, where both the donor and acceptor moieties are emissive semiconductor nanoparticles, as it is necessary that the advantages of this fluorophore choice overcome some of the inherent limitations in QD-QD FRET devices.

A primary advantage of QDs in QD-QD FRET sensing is their extreme photostability compared to organic dyes or fluorescent proteins, making QDs uniquely suited for longitudinal studies, where measurements or images are taken repeatedly over extended periods of time. In addition, the extraordinary brightness of QDs, due primarily to their large absorption cross-section, yields considerable fluorescence output with relatively fewer emitters, potentially lowering the limit of detection in sensing applications. However, QD-QD FRET is challenging due to the broad, overlapping excitation spectra from the two nanocrystals, precluding selective excitation of the donor. This introduces crosstalk and artificially creates a large background signal—a major challenge in QD-QD FRET sensor design. This review provides an overview of the foundational work on QD-QD FRET and discusses published applications. The challenges of achieving efficient QD-QD FRET and how they might be overcome are briefly discussed as well.

## 2. Background

### 2.1. Semiconductor Nanocrystal Quantum Dots

**Figure 1:**
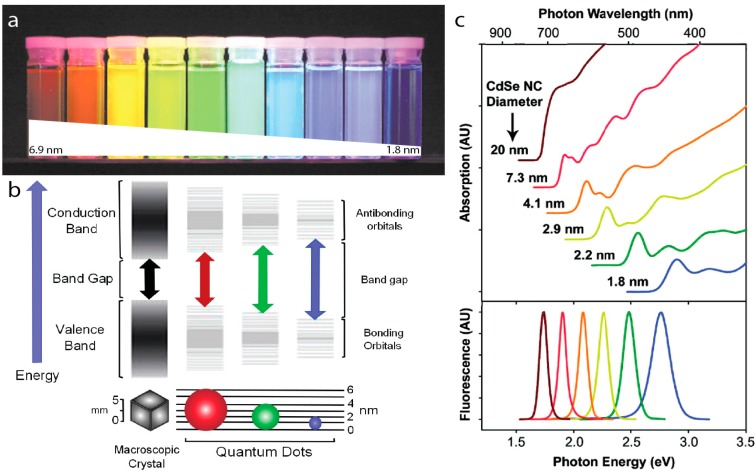
(**a**) CdSe/ZnS core/shell QDs with CdSe core diameters ranging from 6.9 nm to 1.8 nm in diameter emitting with peaks from 1.9–2.8 eV (655–443 nm) from left to right under UV illumination. Adapted with permission from [20]; (**b**) Bandgap energy increases as the nanocrystal size decreases. Reprinted with permission from [38]; (**c**) Absorption (top) and emission (bottom) spectra of CdSe quantum dots. Reprinted with permission from [39]. Copyright (2010) American Chemical Society.

Semiconductor quantum dots (QDs) are crystalline nanoparticles often composed of group II–VI or III–V elements from the periodic table with diameters smaller than their exciton Bohr radius [35], typically just a few nanometers. Quantum confinement effects present at this size range give rise to distinctive optical and electronic properties that are not present in the bulk materials. As the size of the single-crystalline nanoparticle decreases below the exciton Bohr radius of that particular semiconductor, the bandgap—the energy difference between the highest energy valence band and lowest energy conduction band—increases in energy. Since the size of the bandgap dictates the emission energy, the quantum confinement effect ultimately leads to the size-tunable emission of QDs (Figure 1). In addition to size, the bandgap of nanocrystals also depends on its chemical composition. For example, the bulk bandgap of CdSe is 1.74 eV (712 nm) while that of PbS is 0.37 eV (3350 nm). Nanoparticles of CdSe and PbS with decreasing size exhibit increasing bandgaps, which can reach ~3.6 eV (350 nm) [36] and 1.3 eV (950 nm) [15], respectively. CdSe particles with diameters ranging from 2 to 6 nm thus emit photons with energies spanning the visible wavelength range, while PbS QDs emit in the near infrared (NIR). QDs most commonly used in the visible wavelength range are CdSe/ZnS core/shell nanoparticles; the CdSe core confers the particle its unique optical properties, while the ZnS shell serves as a passivation layer, protecting the core from oxidation and enhancing the quantum yield (QY) [19,37].

**Figure 2:**
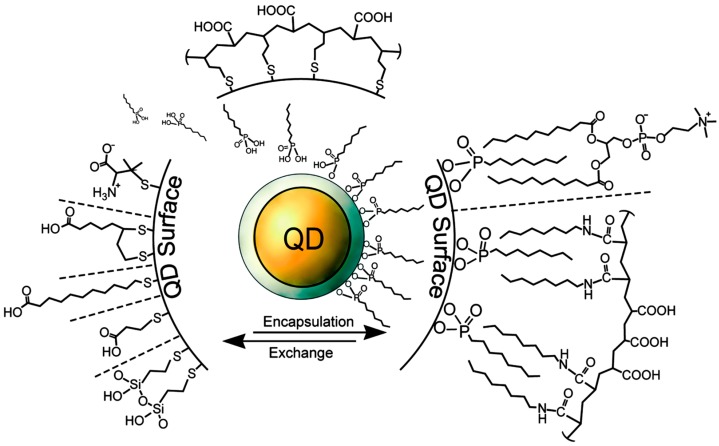
Selected surface chemistries and conjugation strategies, as they apply to QDs. The core/shell NC is depicted in yellow/grey. After synthesis, trioctylphosphene (TOP) surrounds the QD surface (represented by the ligands directly on the grey shell layer). Ligands associate with the QD surface via mono- or bidentate thiols or imidazoles in ligand exchange-based coating schemes and display polar groups or hydrophilic polymers to the media to confer water solubility. Encapsulation strategies utilize the hydrophobic surfactants on the QD surface to facilitate hydrophobic interactions with amphiphilic polymers or lipids. Reproduced with permission from [43].

Colloidal core/shell QDs are manufactured in solution through step-wise injections of organometallic precursors at high temperature. Nucleation and growth are thermally activated and growth continues until arrested by cooling [19]. A layer of coordinating organic ligands surrounds the freshly synthesized core/shell NC, stabilizing the colloid. QDs can be rendered water-soluble by exchanging the hydrophobic surface ligands for charged or hydrophilic moieties (Figure 2) [5]. Typical ligands confer a surface charge for electrostatic stabilization of the NPs or a hydrophilic polymer coating for steric hindrance [40,41,42]. For biosensing applications, one might conjugate oligonucleotides, proteins, or antibodies onto a surface ligand of the quantum dot [5,41]. The ease of tailoring the QD surface functionalities makes them suitable for a wide variety of applications.

### 2.2. Electronic Structure of QDs

Quantum dots have been called artificial atoms due to their discrete energy transitions (Figure 1b), which arise from band splitting due to the quantum confinement effect. The peaks in the absorption spectra (Figure 1c) correspond to the discrete electronic transitions in the QDs [44] and are useful for QD characterization. In particular, the lowest energy absorption peak results from the 1S transition between the lowest energy excited electron in the conduction band and the lowest energy hole in the valence band. The higher-energy transition peaks are seldom used because they are indistinct in non-monodisperse QD populations.

The relationship between the size of the QD, the wavelength of the 1S transition, and the molar extinction coefficient for CdSe, CdTe, CdS, and InP cores have been measured empirically and summarized in a comprehensive review [45]. This enables researchers to determine the size and concentration of these NCs from simple absorption measurements. It should be noted that for some compositions, multiple theoretically- and empirically-based correlations between NC size and spectra have been published, leading to variation in the size and concentration determinations between different papers. In addition, most QD heterostructures do not have well-defined methodologies for determining total core/shell size and molar extinction coefficient or concentration from straightforward absorption measurements. This can present a challenge to researchers when the QD concentration is needed for concerted experimental design.

The 1S peak is used to define the Stokes shift in QDs. Traditionally, the Stokes shift is the difference between the absorption and emission peak maxima [15]. For QDs, the Stokes shift is the difference in maxima between the 1S and emission peaks. Knowledge of the Stokes shift for the QDs of interest enables the determination of the 1S transition from the emission spectra alone, and is useful when the 1S peak is not well defined. The 1S peak and Stokes shift are important features in the optical spectra because they have significant effects on crosstalk within a single population of QDs as well as between QD populations of different sizes (Section 2.4). The Stokes shift is different for different compositions of QDs. For example, CdSe, CdTe, and InP QDs typically exhibit Stokes shifts of 15 nm, 35 nm [46], and 55 nm [47,48], respectively. The Stokes shift can also be affected by doping, as is thoroughly reviewed in [49].

Depending on the bandgap alignment of the donor and acceptor, core/shell QDs exhibit different photophysical properties, as is thoroughly reviewed in [45]. Briefly, the most common bandgap alignments result in Type I, Type II, and Quasi-Type II QDs. In Type I core/shell QDs, the bandgap of the core is smaller than that of the shell with the lowest energy level for both the conduction and valance bands appearing in the core. As the excited electron and hole of an exciton both relax to their lowest energy states, the exciton is confined to the QD core, and the core bandgap alone dictates the emission energy. In a Quasi-Type II heterostructure, the hole remains confined in the core, but the energy levels of the core and shell conduction bands are close enough that the excited electron can reside in either, resulting in a spreading of the electron from the core into the shell. In this structure, the emitted photons are red-shifted compared to the isolated core and the fluorescence lifetimes are somewhat longer. In a Type II structure, the core and shell bandgaps are staggered, so that the electron and the hole are spatially separated in the shell and core, respectively. The resulting recombination is dramatically red-shifted compared to the core, and can be lower in energy (redder) than the bulk bandgap of the core material. In addition, the spatial separation of the electron and the hole results in a much longer fluorescence lifetime (hundreds of nanoseconds) and considerably lower QY compared to Type I and Quasi-Type II heterostructures.

For decades, semiconductor cores have been passivated with a monolayer or two of a semiconductor shell to enhance the QD optical properties and chemical stability [19], especially for applications that require water-soluble NPs. More recently, ultra-thick shells (10–20 monolayers) have been added to cores, producing so-called ‘giant’ nanocrystal quantum dots (g-NQDs) that exhibit improved chemical robustness and interesting photophysical advantages, such as suppressed blinking [50,51]. The addition of the thick shell means that the absorption profile of the NC shows a strong increase in the absorption cross-section at high-energy wavelengths (bluer than the bulk bandgap of the shell semiconductor). The use of novel shell compositions and thicknesses may enable the absorption spectrum of the NCs to be tailored much in the way that the emission color is tailored by the physical structure of core QDs.

**Figure 3:**
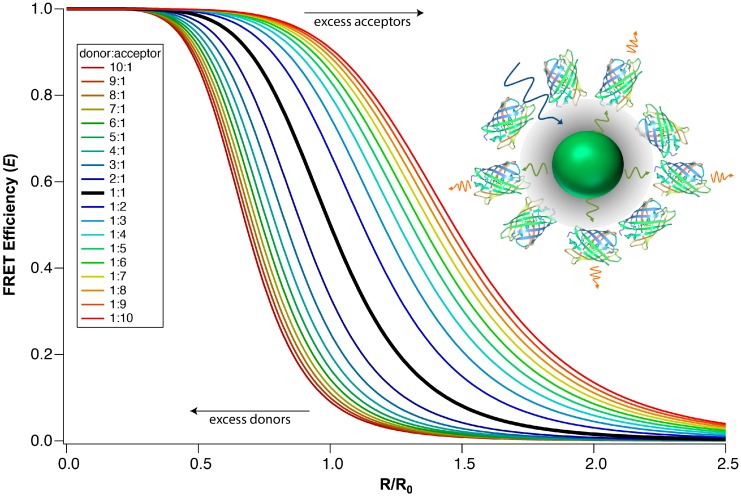
The distance dependence of FRET efficiency for multivalent systems. The FRET efficiency at a given distance improves as the number of acceptors per donor, **n**, increases, and *vice versa*. The inset shows an example of a multivalent system: multiple fluorescent protein acceptors are bound to a single QD donor. Inset reprinted with permission from [47]. Copyright (2012) American Chemical Society.

### 2.3. FRET Equations

Resonance energy transfer between quantum dots follows the framework proposed by Förster in 1948. Förster theory can be summed up in three equations [1,2,52]. First, the energy transfer efficiency, *E* (or η_FRET_, as presented in some sources [53]), is traditionally described as:

(1) $$E=\frac{R_{0}^{6}}{R_{0}^{6}+R^{6}}$$

where *R* is the distance between donor and acceptor, and *R*_0_ is the Förster distance, *i.e.*, the donor-acceptor distance at which FRET efficiency (*E*) is 50%. In some scenarios, a donor is in close proximity to many acceptors or *vice versa*. It has been shown that FRET efficiency increases with an increasing number of acceptors per donor. A modified transfer efficiency equation is used to take into account the multivalency in such systems [22,53]:

(2) $$E=\frac{nR_{0}^{6}}{nR_{0}^{6}+R^{6}}$$

where **n** is the number of acceptors per donor. Intuitively, as more acceptors are present, more energy transfer pathways are available, and the probability of de-excitation via FRET becomes greater. Experimentally, it has been clearly shown that the FRET efficiency in QD-dye and QD-FP FRET systems scales with respect to Equation (2) when the donor-acceptor distance is held constant and the ratio of donor and acceptor molecules is varied [22,23,54]. Figure 3 demonstrates how an increase in the number of acceptors per donor increases the transfer efficiency. The impact of multivalency on FRET efficiency enables complex FRET systems to achieve a higher *E* than traditional single-donor-single-acceptor systems. Figure 3 also demonstrates that the transfer efficiency drops prodigiously if there are fewer acceptors than donors (*i.e.*, **n** < 1).

The transfer efficiency, or the probability of de-excitation via energy transfer, can also be expressed in terms of the donor lifetime or emission intensity [2]:

(3) $$E=1-\frac{{\text{τ}}_{DA}}{\tau_{D}}=1-\frac{F_{DA}}{F_{D}}$$

where τ*_DA_* and τ*_D_* are the lifetime of the donor in the presence and absence of an acceptor, respectively. *F_DA_* and *F_D_* are the fluorescence intensity of the donor in the presence and absence of the acceptor, respectively. From these equations, we can see that the decrease in donor intensity and lifetime are indicative of increased FRET efficiency.

Finally, the efficiency can be related to the rate of the resonance energy transfer, *k_T_* [2]:

(4) $$E=\frac{k_{T}}{k_{T}+k_{D}}=\frac{k_{T}}{k_{DA}}$$

where *k_D_* is the rate of total donor luminescence decay (*k_D_* = *k_D,rad_* + *k_D,nonrad_*). This equation describes the energy transfer efficiency as the fraction of energy transfer via FRET versus total energy loss from the donor in the presence of the acceptor, *k_DA_*, which includes FRET, radiative losses, and non-radiative losses. Isolating *k_T_* and substituting Equation (1) for *E* yields the equation for the rate of energy transfer [2]:

(5) $$k_{T}=\frac{1}{{\text{τ}}_{D}}\left(\frac{R_{0}^{6}}{R^{6}}\right)=\;\frac{1}{{\text{τ}}_{T}}$$

The FRET rate, *k_T_*, is reported as (τ*_T_*)^−1^, and a shortened donor lifetime in the presence of an acceptor correlates to faster and more efficient energy transfer [12,55,56].

The Förster distance, *R*_0_, mentioned above is a characteristic value that can be determined for each donor-acceptor pair using the following formalism [2]:

(6) $$R_{0}^{6}=\frac{9\left(ln10\right){\text{κ}}^{2}{\text{Φ}}_{D}}{128{\text{π}}^{5}n^{4}N_{A}}J$$

where *Φ_D_* is the quantum yield of the donor fluorescence in the absence of the acceptor, *n* is the index of refraction of the medium, *N_A_* is Avogadro’s number, *J* is the overlap integral, and *κ^2^* is the dipole-dipole orientation factor. κ*^2^* can range from 0 to 4 and is often assumed to be 2/3 as this value corresponds to isotropically oriented dipoles, as a result of freely rotating donor and acceptor molecules. As *R*_0_ represents the donor-acceptor distance that yields 50% FRET efficiency, one can compare the relative strengths of various FRET pairs by comparing their Förster distances: pairs with longer Förster distances exhibit higher FRET efficiencies than pairs with shorter Förster distances under the same conditions.

**Figure 4:**
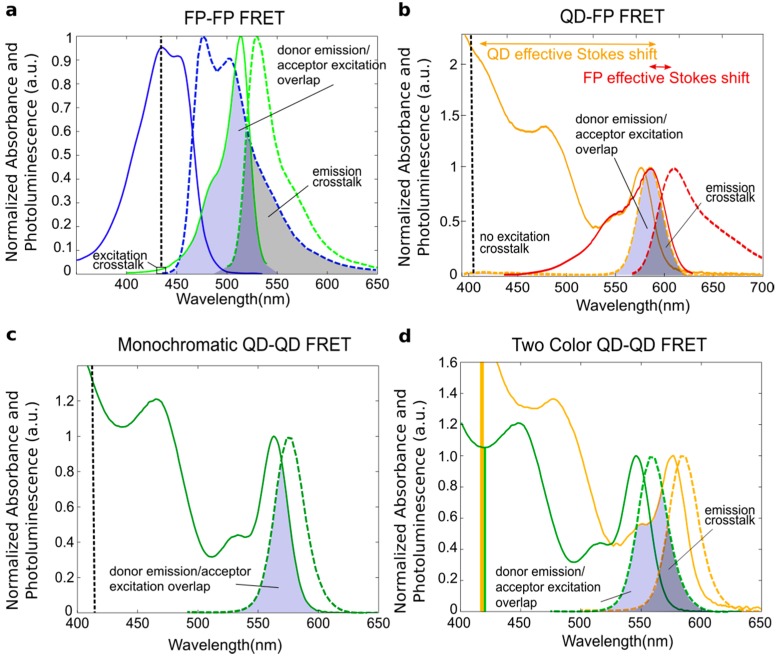
Normalized excitation and emission spectra of four different FRET pairs with schematized spectral overlaps shown. (**a**) Enhanced cyan fluorescent protein (ECFP, blue, donor) and enhanced yellow fluorescent protein (EYFP, green, acceptor); (**b**) A 3.7 nm CdSe/ZnS QD (orange, donor) and the fluorescent protein mCherry (red, acceptor); (**c**) A 3.3 nm diameter CdSe/ZnS QD acting as both donor and acceptor; (**d**) A 2.9 nm diameter CdSe/ZnS QD (green, donor) and 3.7 nm diameter CdSe/ZnS QD (yellow, acceptor). The vertical dotted black line in (a,b,c) indicates the expected excitation wavelength with the box indicating excitation crosstalk (*i.e.*, where both the donor and the acceptor absorb the excitation light); In (d), the excitation crosstalk is represented with yellow and green bars, demonstrating that the acceptor has a significantly larger absorption than the donor, making excitation crosstalk a significant issue. The spectral overlap between the donor emission and acceptor excitation is shaded blue; the emission crosstalk (*i.e.*, overlap between the donor and acceptor emission) is shaded grey. (a,b) were made using FP spectral data from [58].

The overlap integral, *J*, is the integral of spectral overlap between the donor emission and the acceptor absorption multiplied by wavelength to the fourth power [2]:

(7) $$J=\int_{0}^{\infty }f_{D}\left(\text{λ}\right){\text{ε}}_{A}\left(\text{λ}\right){\text{λ}}^{4}d\text{λ}$$

where *f_D_*(λ) = *F_D_*(λ)/∫*F_D_*(λ)*d*λ is the donor emission spectrum normalized to its area, and *ε_A_*(λ) is the molar extinction coefficient of the acceptor. The in-depth derivation of *J* as well as the rest of Förster theory can be found in [57]. When calculating *J*, special care must be taken to normalize the spectra appropriately and carefully account for the differing units in the various terms, as described in [2,53], to ensure the proper determination of *J* and thus *R*_0_. The blue-shaded region in Figure 4 illustrates the spectral overlap, but it is not the integrand of *J*. The actual integrand of *J* will not resemble the spectral overlap due to the contribution from λ^4^.

Table 1 provides a summary of the optical properties of the donors and acceptors depicted in Figure 4 as well as the calculated *J* and *R*_0_ values for the FRET pairs. The overlap integral and Förster radius calculated for the ECFP-EYFP pair in Figure 4a is in a typical range for FP-FP pairs [59]. Using a QD donor that is perfectly color matched to the FP acceptor, as in Figure 4b, results in a larger overlap integral and Förster radius. The increased acceptor molar extinction coefficient when the QD acceptor is used leads to a further increase in *J* and *R*_0_; optimizing the spectral overlap of the QD donor and acceptor in hetero-FRET leads to the largest values of the four systems. The high molar extinction coefficients of the donor QDs at the excitation wavelength increase the brightness of the QD-based systems significantly, but the high excitation wavelength cross-sections of the acceptor QDs also lead to high background due to direct acceptor excitation.

**Table 1:** Optical properties for the FRET pairs depicted in Figure 4.

	FRET Pair	Donor Molar Extinction Coefficient (ε*_D_*; M^−1^∙cm^−1^)	Acceptor Molar Extinction Coefficient (ε*_A_*; M^−1^∙cm^−1^)	Donor Quantum Yield (*Φ_D_*)	Overlap Integral (*J*; M^−1^∙cm^−1^∙nm^4^)	Förster Distance (*R*_0_; nm)
a	FP-FP	32,500 ^a^ (ECFP)	83,400 (EYFP)	0.40	1.99 × 10^15^	4.53
b	QD-FP	190,860 (λ_1S_); ^b^ 389,700 (λ_e_) ^c^	72,000 (mCherry)	0.60 ^d^	6.20 × 10^15^	5.86
c	QD-QD (Homo-FRET)	142,220 (λ_1S_); 208,800 (λ_e_)	142,220 (λ_1S_); 208,800 (λ_e_)	0.60	8.52 × 10^15^	6.18
d	QD-QD (Hetero-FRET)	102,370 (λ_1S_); 116,200 (λ_e_)	190,860 (λ_1S_); 387,900 (λ_e_);	0.60	1.29 × 10^16^	6.63

^a^ FP optical property values from [60]; ^b^ QD molar extinction coefficients calculated at the 1S peak wavelength using the equation listed in [45]; ^c^ QD molar extinction coefficients calculated as a possible excitation wavelength (405 nm) using the 1S molar extinction coefficient and the measured absorbance spectrum; ^d^ QD quantum yield set at a 60% as a typical QY reported by multiple groups producing CdSe/ZnS core/shells, as summarized by [45].

The energy transfer efficiency and energy transfer rate can be increased by increasing *R*_0_ and decreasing *R*. Methods of decreasing *R* in QD-QD systems include increasing the concentration of donors and acceptors in solution, inducing specific binding or non-specific aggregation of donor and acceptor NPs, and decreasing the size of QD surface ligands to reduce the spacing between associated donors and acceptors. However, there is a limitation to this scheme: Förster theory is only valid when donors and acceptors are within each other’s near-field zone, usually between 1 and 10 nm [52]. More specifically, 0.01b~0.1b nm, where b = λ/2π*n*, λ is the donor fluorescence wavelength, and *n* is the index of refraction of the solvent. As *R* < ~1 nm, acceptors enter the contact (or Dexter) zone of the donor, and the ideal dipole approximation, on which Förster theory is based, breaks down [52,57]. In this close proximity, complex formation and Dexter Electron Transfer becomes likely, and the probability of exciton relaxation via FRET becomes much lower. This limitation of Förster theory and its implications are discussed in detail in [57]. Because of the size of the NC and the surface coatings used to maintain colloidal stability, however, it would be a challenge to design a QD-based construct that yielded a donor-acceptor distance of less than one nanometer. This eliminates the limitations of Förster theory as a concern in most sensor designs.

The most direct way to increase *R*_0_ is to maximize the overlap integral, *J*, by carefully selecting donors and acceptors such that the absorption and emission spectra maximally overlap. In addition, selecting donors with high quantum yields and acceptors with considerable molar extinction coefficients increases the Förster distance and spectral overlap, respectively. Approaches that increase the molar extinction cross-section of the donor also increase the overall light output of the system and the amount of energy transferred, without directly increasing the FRET efficiency. For example, light harvesting antenna (Section 5) can be used to increase the absorption cross section of QD donors [61,62,63], thereby increasing the overall number of photons absorbed and the total amount of energy transmitted, independent of energy transfer efficiency.

### 2.4. Crosstalk

FRET between two organic fluorophores has long been used in fluorescence microscopy [2]. However, there are limitations to using organic fluorophores, including susceptibility to photobleaching and oxidative degradation, short fluorescence lifetime, and spectral crosstalk [15]. Crosstalk occurs when a signal from the donor and acceptor are both present in a relevant wavelength range, resulting in a significant background signal. There are two types of crosstalk: excitation and emission crosstalk. Excitation crosstalk occurs when there is an overlap in the excitation spectra of the donor and acceptor. In this case, direct acceptor excitation (*i.e.*, acceptor emission due to the absorption of photons from external illumination) and sensitized emission (*i.e.*, FRET-based emission) from the acceptor cannot be distinguished without proper controls. Emission crosstalk occurs when the emission spectrum of the donor overlaps with the emission spectrum of the acceptor, thus producing background signal in the acceptor emission channel (Figure 4). Analytical and image processing methods exist to account for crosstalk in both spectral data and fluorescence microscopy images, but the methods require much more stringent experimental design and analysis than is necessary for fluorophores that are not susceptible to crosstalk [64,65].

QDs exhibit broad absorption spectra and narrow emission spectra, producing a large energy shift between the high-energy absorption states and the emission band. This generates a large effective Stokes shift in addition to the more typical Stokes shift between the 1S absorption peak and the emission band (Figure 4b). For this reason, crosstalk, or spectral bleed through, is less of an issue in QD-dye and QD-fluorescent protein hybrid systems compared to FRET systems utilizing two organic fluorophores, as ultraviolet (UV) illumination can be used to excite the QD far from the acceptor absorption. Excitation crosstalk is a significant issue in QD-QD FRET systems, however, where high-energy excitation (*i.e.*, UV illumination) directly excites both the donor and acceptor (Figure 4d). Amongst QDs of the same composition, larger, redshifted acceptor QDs will in fact exhibit larger absorption cross-sections at the excitation wavelength than the donor QD (Figure 4d). This causes a large background signal due to direct acceptor excitation, which has to be distinguished from sensitized emission (emission due to energy transfer).

Looking at the QD spectra, one can see that in a nominally monodisperse and monochromatic QD population, crosstalk exists between the absorbance and emission spectra, yielding a non-zero spectral overlap (*i.e.*, *J* ≠ 0) (Figure 4c). This results in energy transfer within a nominally monochromatic population of QDs if the distance between QDs is within the FRET range (nominally, 0.5*R*_0_ < *R* < 2*R*_0_).

### 2.5. FRET Measurement Techniques

**Figure 5:**
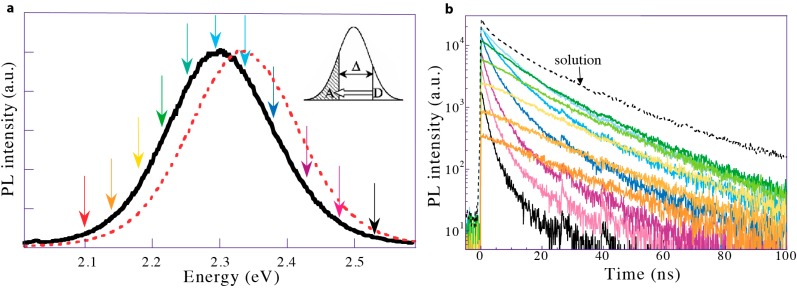
(**a**) FRET within a nominally monochromatic QD population. Higher energy QDs donate energy to lower energy QDs in the same inhomogeneous population. The PL spectrum of the QDs in solution (dotted red line) redshifts and narrows slightly when the QDs are in a dense film (solid black line); (**b**) Time-resolved PL measured at energies corresponding to the colored arrows in (a). The black dotted trace shows the time-resolved PL of the QDs in solution. At high energies, QDs in a dense film exhibit multiexponential PL decay; the rapid decay component dominates as energy is siphoned off donors through FRET. At lower energies the intensity decays less slowly than in solution as the acceptor QDs receive an influx of energy after the excitation pulse from FRET. Adapted with permission from [56].

Equation (3) enables the calculation of FRET efficiency from experimental data, namely fluorescence intensity and lifetime. Fluorescence intensity is measured with a spectrometer, and a typical plot of the resulting spectrally resolved photoluminescence is shown in Figure 5a. The PL data is obtained by exciting the molecule at one specific wavelength then measuring its fluorescence over a range of wavelengths. It is represented as the PL intensity versus either energy (eV) or wavelength (nm). Spectral evidence of FRET comes from the red-shifting of the emission peak in monochromatic FRET or the decrease in the donor emission intensity and an increase in the acceptor emission intensity in two-color FRET systems. Fluorescence lifetime is calculated from the time-resolved photoluminescence spectra (Figure 5b). Time-resolved PL spectrographs display the histogram of photons emitted at discrete times following pulsed excitation as measured at a particular wavelength. A curvefit to this plot is used to calculate the average fluorescence lifetime of the fluorophore. In the presence of FRET, the donor average lifetime decreases as the fast-acting energy transfer siphons off photonic energy. Concomitantly, the acceptor average lifetime increases as it receives a post-excitation-pulse influx of energy.

## 3. QD-QD FRET

Though FRET can occur between any fluorophores exhibiting spectral overlap between the donor emission and acceptor excitation, it manifests differently depending on the population of fluorophores present. Homotransfer, or energy transfer between multiple copies of the same molecule, is a well-known phenomenon for organic fluorophores and has been studied both in ensemble and single-molecule experiments [2,66]. Heterotransfer is the energy transfer between two different species (two fluorophores or a fluorophore and a quencher) and has been used extensively in sensing applications [2,53].

### 3.1. Homotransfer amongst QDs

In contrast to organic (*i.e.*, molecular) fluorophores, individual QDs have discrete atom-like energy spectra. A single quantum dot of a specific size emits at a discrete wavelength that corresponds to its bandgap, as would an ensemble of identically sized QDs [67]. Thus, if one were to consider the energy transitions between two identical QDs, spectral overlap, and thus homotransfer, is unlikely. In an ensemble, however, one observes both inhomogeneous and homogeneous peak broadening, and therefore homotransfer. Temperature-independent inhomogeneous broadening is an effect of the variations in the particle size, shape, composition, etc., within the sample. Temperature-dependent homogeneous broadening is observed even within a perfectly monodisperse ensemble due to exciton-phonon interactions, with broader peaks being observed at higher temperatures [68]. Although QD monodispersity can be increased post-synthesis by size-selective precipitation [69] and synthesis methods have evolved to improve the as-synthesized batch monodispersity [70,71,72], it is still exceedingly difficult to obtain a perfectly monodisperse NC population. As a result, a nominally monodisperse QD population contains a distribution of sizes, corresponding to an emission peak with a Gaussian distribution.

In QDs, homotransfer takes place in a nominally monochromatic population [55,56,73,74,75,76,77,78,79,80], where there is a large spectral overlap between the luminescence spectra of slightly smaller donor QDs and the absorption spectra of subtly larger acceptor QDs. As a result, homotransfer is exhibited by a quenching of the blue luminescence and an enhancement of the red luminescence within the monochromatic peak, causing the emission peak to redshift and the emission linewidth to narrow (Figure 5a) [75]. In addition to this spectral shift, a FRET-induced change in the donor and acceptor PL lifetimes is evident. The donor average lifetime decreases, while the acceptor average lifetime increases [56,75,77,81].

A thorough theoretical analysis of the impact of inhomogeneous peak broadening on energy performed by Kagan *et al*. demonstrated that the spectral signature of FRET should be discernible in samples with size deviations as small as 1.5%, and FRET efficiency should improve with increasing size dispersity [81]. Crooker *et al*. presented a dynamical study on FRET between CdSe QDs that discusses the positive correlation between the FRET efficiency and increased QD size distribution. Since efficient coupling consists of a ground-state exciton in a donor in resonance with a strong absorption transition in a nearby acceptor, only dots sufficiently larger than the donor serve as efficient acceptors (Figure 5a) [56]. In monochromatic CdSe QDs, Crooker *et al*. modeled the PL decay rate as a function of energy and showed that energy transfer only occurs if acceptors have a bandgap smaller than the donors by at least 55 meV [56]. This approach was adopted by Bose *et al*. who applied it to studying monochromatic PbS populations and observed energy transfer only when the acceptor bandgap was 100 nm larger than the donor bandgap (for QDs emitting in the NIR, ca. 1400 nm, this corresponds to a donor-acceptor energy difference of ~59 meV) [55]. This energy separation requirement stems from the separation between the donor emission spectra and the acceptor absorption 1S peak. Lunz *et al*. also observed that as the QD size distribution broadens, a larger proportion of the QD population meets this requirement, and FRET becomes more efficient [46].

**Figure 6:**
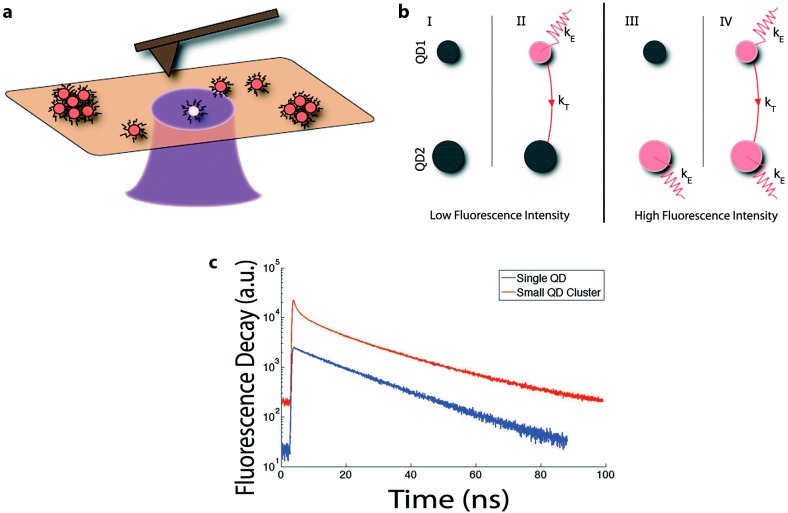
(**a**) Schematic of correlated fluorescence and atomic force microscopy on single QDs and QD clusters; (**b**) Schematic of possible emission and energy transfer schemes accounting for independent blinking (on/off states) of a QD donor (QD1) or acceptor (QD2); (**c**) Time-resolved PL of a single QD and a small QD cluster. Schematics are not drawn to scale. Reprinted with permission from [77]. Copyright (2010) American Chemical Society.

FRET within a monochromatic population can take place in any species of QDs. Mayilo *et al*. [76] and Tang *et al*. [78] report on FRET within colloidal CdTe and nanochains of CdTe QDs, respectively. Several groups have also observed the characteristic redshift in PL spectra as well as decreased donor PL lifetime when reporting on homo-FRET in PbS [55,73,74] and InP [80] populations. A particularly elegant homotransfer study by Shepherd *et al*. examined single CdSe/ZnS QDs and small clusters (2–10 QDs per cluster) with coordinated single-molecule fluorescence and atomic force microscopy (AFM) techniques [77]. With this added resolution, it was shown that the time-resolved PL of a single CdSe/ZnS QD decays monoexponentially, modeled by *D_single_*(*t*) = *Ae^t/^*^τ^, while that of clustered QDs is better modeled by a biexponential [77]:

(8) $$D_{FRET}\left(t\right)=Ae^{-t/{\text{τ}}_{1}}+Be^{-t/{\text{τ}}_{2}}$$

where τ_1_ and τ_2_ are the time constants (lifetimes) of the short- and long-lived components, respectively. The short-lived component describes the rapid PL decay due to energy transfer, while the long-lived component describes the steady-state PL decay without energy transfer (Figure 6). Equation (8) is not limited to homo-FRET. In general, the donor lifetime is usually found by fitting Equation (8) in time-resolved PL spectra of the donors.

By studying isolated clusters, Shepherd *et al*. were able to address the impact of fluorescence intermittency, or blinking, on energy transfer. Several combinations of on and off states are possible for a two-dot donor/acceptor scenario, leading to low and high fluorescence states (Figure 6b). One can see that if the acceptor is in an “on” state (cases III and IV), the system is highly emissive from either direct acceptor excitation and/or FRET-based sensitized emission. If the acceptor is in an “off” state, the system is overall less emissive, regardless of whether energy transfer is taking place or not (cases I and II). This demonstrates why some highly concentrated QD samples exhibit self-quenching (*i.e.*, concentration quenching or inner filter effect [2]) as non-emissive acceptors siphon off energy (II). This scheme also clarifies why total emission from a system is not a strong indicator of FRET, as emission can decrease with or without energy transfer (I and II). Likewise, acceptor emission intensity is not sufficient to demonstrate FRET, as it can be confounded by emission due to direct excitation. Additionally, changes in acceptor emission intensity could come from energy transfer or any condition that alters the on/off state of the QDs. In contrast, the donor PL, either spectral intensity (in the two-colored systems discussed below) or the corresponding changes in fluorescence lifetime, does appropriately indicate energy transfer from on-state donors, regardless of the state of the acceptor. This is consistent with Equation (3), where it is shown that energy transfer efficiency is measured by examining only the donor intensity or lifetime, thereby ignoring what might happen to the transferred energy once the acceptor or quencher receives it.

In addition to blinking and the inner filter effect, quenching can also be caused by homotransfer within QD populations. In ensemble measurements, self-quenching of the fluorophore through homo-FRET is evident at high local fluorophore concentrations as the probability that energy is transferred to non-emissive fluorophores that act as an energy sink increases [66]. Using species with a larger Stokes shift can mitigate this effect. As the emission peak shifts further away from the 1S peak, the overlap integral between the peaks decreases and less homotransfer will take place [46]. Although no direct comparison was found in the literature, this relationship indicates that QD compositions exhibiting smaller Stokes shift, like CdSe, are likely more prone to homotransfer, while large Stokes shift compositions, such as InP, may exhibit less efficient homo-FRET and could potentially be more effective donors in two-color systems.

Red-shifting within a single QD population is spectrally subtle, occurring over several meV (Figure 5). In addition, the lifetime of acceptor QDs is approximately equal to that of non-interacting QDs [56], indicating that the sensitized emission from FRET is relatively weak. In other words, the acceptor QD emission is still overwhelmingly dominated by direct excitation. The subtle effect of homotransfer FRET in monochromatic QD populations makes it non-ideal for sensing applications.

### 3.2. QD-QD Heterotransfer

**Figure 7:**
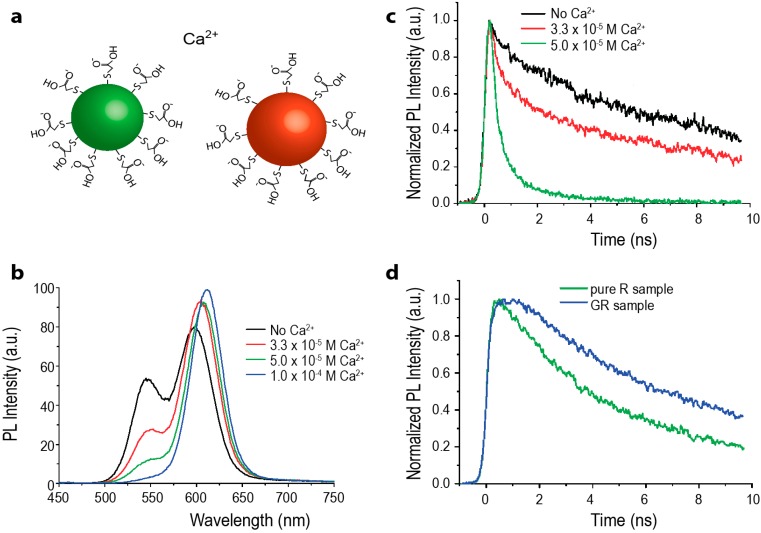
(**a**) Schematic of two-color QD-QD FRET Ca^2+^ sensor. Green donor CdTe QDs and red CdTe acceptor QDs are each coated with thioglycolic acid (TGA), imparting a negative surface charge. In the presence of the calcium cation, the QDs aggregate, bringing them in close enough proximity for energy transfer to occur efficiently. Schematic is not drawn to scale; (**b**) The PL spectrograph shows a decrease in the green donor emission and increase in the red acceptor emission with increasing calcium cation concentration; (**c**) Time-resolved PL of the green emission with increasing amounts of calcium; as the cation concentration increases, QD-QD interactions are promoted. The donor emission lifetime visibly shortens, indicating that the green QDs are acting as FRET donors; (**d**) Time-resolved PL of deep red emission from red-only and green-and-red-mixed QD samples. In the presence of the green donor QD, the PL lifetime of the red emission is elongated; (b–d) reprinted with permission from [76]. Copyright (2008) American Chemical Society.

Similarly to monochromatic QD populations, FRET is observed amongst differently sized QDs of the same species, *i.e.*, heterotransfer between two spectrally distinct, but physically co-mingled, QD populations. In a mixed population of QDs, the smaller, higher energy NCs act as donors while larger, lower emission energy NCs act as acceptors. When FRET occurs, the donor emission is quenched and the acceptor emission is enhanced (Figure 7b).

As in monochromatic populations, FRET between mixed-sized QDs can occur in any species of QDs. The earliest report on heterogeneous QD-QD FRET was by Kagan and coworkers [75,81], and followed by examples of FRET in heterogeneous populations of CdSe [82], CdTe [83], PbS [74], InP [69], and ZnO [84]. These reports typically describe the characteristic ratiometric shift in overall PL spectra, decreased donor lifetime, and increased acceptor lifetime indicative of FRET (Figure 7). Ratiometric FRET signals from heterogeneous QD samples are better suited for sensing applications than the spectral output from monochromatic QD FRET, because the color and intensity changes are much more prominent. Despite this much clearer spectral change, direct acceptor excitation due to spectral crosstalk is still a significant limiting issue, as lower-energy acceptor QDs are larger than the donor QDs and thus exhibit higher absorption cross-sections.

### 3.3. FRET between Different Species of QDs

Very few reports of FRET between heterogeneous QDs of different species have been published, but those few examples explore the potential for FRET between bandgap engineered QDs. For example, Wang *et al*. demonstrated FRET between CdSe/ZnS (Type I) and CdSe/ZnTe (Type II) QDs [85]. This construct illustrates how the same core (CdSe) with two different shells (ZnS or ZnTe) results in QDs with significantly different optical properties that can be used in different roles in the FRET device. The Type I donor has a high QY (0.7) and shorter fluorescence lifetime while the Type II acceptor has a very low QY (0.01) and longer fluorescence lifetime. The Type I QDs acted as FRET donors even though they have shorter fluorescence lifetimes than the long-lived Type II acceptors. Recall that organic dyes are known to be ineffective FRET donors to QD acceptors precisely because of the difference in the fluorescent lifetimes of the donor and acceptor, and that longer-lived donors are preferred [28]. Although the fluorescent lifetimes of the donor and acceptor QDs are not specifically reported in Wang *et al*., it appears that the difference in the lifetimes of the QD species is not as dramatic as between a QD and organic dye, thus allowing some measurable amount of energy transfer. Despite the shorter donor lifetimes, there are two reasons why the Type I QD must be the donor to the Type II acceptor: (1) using the same core, the Type II NC will always emit redder than the Type I NC; (2) Type I QDs typically exhibit high QYs, as is necessary for efficient energy transfer, while Type II QDs typically exhibit prohibitively low QYs.

Another group solved the problem of the shorter donor lifetimes by using Cu- and Mn-doped d-dots (doped QDs) with fluorescent lifetimes in the micro- and milli-second range as donors to CdSe QD acceptors. Although the d-dots and QDs were not actively bound to one another, making the donor-acceptor distance unclear, the authors did observe spectral overlap-dependent changes in the donor and acceptor emission intensities, indicating energy transfer [86]. While these experiments were not performed in a way that fully elucidates the limits and benefits of interspecies QD-QD FRET, they do at least hint at the way that the donor and acceptor lifetimes, spectral overlap, and excitation spectra may be tailored in a concerted way by utilizing donor and acceptor NCs of unrelated compositions in order to generate the optical properties that are best suited to the role of those materials in the device.

Just as the photophysical studies into interspecies QD-QD FRET are still immature, very few examples of this kind of energy transfer process being used in applications. In the only notable study, Ebenstein *et al*. cleverly took advantage of the *R*^6^ dependence of FRET and applied it to AFM [87]. The tip of the AFM was functionalized with InAs QDs, which quenches (or enhances) the PL signal from CdSe/ZnS QDs as a function of distance. They showed that distance-dependent fluorescence quenching scheme enabled them to achieve high-resolution near-field imaging. Although more theoretical studies are needed to fully utilize this configuration in an AFM setup, the data presented so far is promising.

### 3.4. Controlling Donor-Acceptor Distance through Concentration

Due to the 1/*R*^6^ dependence of FRET, the effects of FRET grow exponentially more pronounced as the interdot distance decreases. QDs can be brought into close proximity by increasing the QD concentration either locally or globally. Binding QDs to each other (whether specifically or non-specifically) changes the local concentration within a solution [88,89], while the evaporation of solvents increases the QD concentration globally [90,91], although somewhat asymmetrically. For example, drop-cast QDs have shown macroscopic morphological variability, such as a coffee-ring pattern, indicating a higher concentration of particles around the edge of the drying drop of QDs in volatile solvent [92]. While the interdot spacing cannot be precisely controlled during solvent evaporation, the ensemble QD concentration and interdot spacing are clearly changing with time, making it possible to study the dynamics of FRET during this process [90,91]. Xu *et al*. present an equation for approximating the average interdot spacing in a solution [91] (incorrectly cited by [90]):

(9) $$\frac{V}{N_{QD}}=\frac{4}{3}\text{π}r^{3}=\frac{1}{6}\text{π}R^{3}$$

where *V* is solution volume, *N_QD_* is the number of QDs in solution, *r* is the radius of the sphere that a single QD occupies, and *R* = 2*r* is the mean center-to-center distance between two QDs, or the donor-acceptor distance [90]. *N_QD_* can be estimated from the QD absorption spectra using published molar extinction coefficients for particular QD core compositions (for example, [93]), but this is limited to specific compositions within specific size ranges, is somewhat error prone, and becomes more challenging for complex heterostructures [94], if published values exist for the particular heterostructure at all.

**Figure 8:**
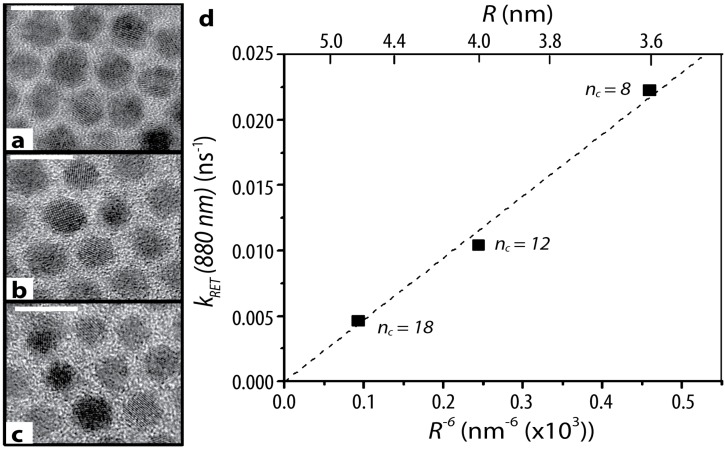
PbS dots with (**a**) C8 (**b**) C12 and (**c**) C18 ligands corresponding to average surface-to-surface separations of 1.0 ± 0.2, 1.4 ± 0.5, and 2.1 ± 0.5 nm, respectively. Scale bars are 10 nm on all images; (**d**) The rate of non-radiative resonance energy transfer (RET) as a function of the sixth root of the interdot distance with the corresponding center-to-center interdot distance on the upper axis. Reproduced with permission from [73]. Copyright (2011) American Chemical Society.

The complete evaporation of the solvent results in a thin film of QDs. In QD films formed this way, the interdot surface-to-surface distance is dictated by the size of capping ligands on the QD surface [73]. Lingley *et al*. confirmed this by observing that the rate of resonance energy transfer between neighboring PbS QDs in drop-cast films is linearly proportional to the sixth root of the donor-acceptor distance (*R*^−6^) (Figure 8). The interparticle distance was adjusted by changing the ligand on the surface of the QD, ranging from eight to eighteen carbons in the hydrocarbon chain of the capping ligand, and precisely measured with TEM [73].

Evaporating the solvent is a simple way increase the QD concentration in solution, and is an easy way to induce FRET in a system. One caveat, however, is that the rate of evaporation is difficult to control, making it challenging to control the QD concentration. It has also been observed in the evaporation studies that QDs in solution are often too sparse to induce FRET; it is not until they reach the “gel-phase” that the interdot distances become small enough for FRET to occur [90,91].

### 3.5. Controlling Donor-Acceptor Distance by Embedding QDs in Polymers

Interdot spacing can be modified by embedding QDs into a support matrix [79,95,96]. Chen *et al*. studied the effects of FRET between two different sizes of CdSe QDs by depositing them onto PDMS films; the interdot distance could be reversibly changed by stretching the film [95]. Similarly, Xu *et al*. controlled the interdot distance of TGA-capped CdTe QDs embedded in a gelatin film by controlling the amount of gelatin present in the film, where a higher gelatin content created a larger interdot distance [79]. Note that due to the presence of spacer materials, the minimum interdot distance is larger than that of QD films, where QDs can aggregate directly with one another. On the other hand, the color changes from FRET are directly visible in the gelatin films, making for a compelling visual display of the effects of FRET at different interdot distances [79]. Although these methods have limited use as sensors, these studies successfully induced FRET and presented unique methods to probe the physical properties of QDs and the photophysics of QD-QD FRET. Recently, Generalova and coworkers made a temperature sensor that is sensitive between 20 and 40 °C by embedding QDs into a temperature sensitive polymer [96]. As the temperature decreases and the polymer shrinks, QDs are brought closer together, resulting in more homo-FRET. This causes more quenching of the photoluminescence with a decrease in temperature, and *vice versa*. This clever sensor using QD-QD homotransfer could be used to visually monitor the temperature in micro-scale environments, where traditional probes cannot reach.

QDs can similarly be embedded into polymers for more rigid amorphous composites. There are examples in the literature of QDs being embedded in poly(methyl methacrylate) (PMMA), poly(lauryl methacrylate) (PLMA), and polystyrene (PS) [68,97]. As with solution-phase experiments, if the concentration (locally or globally) of the QDs is high enough in the polymer film/solid, then FRET can be observed. Of these materials, PLMA in particular has the advantage of a long hydrophobic tail in the repeated portion of the polymer structure, which is believed to interact with the hydrophobic surface coating on the QDs, effectively stabilizing the colloid prior to polymerization. This stabilizing interaction can mitigate local cluster formation or phase separation of the QDs during polymerization, yielding a “solid solution” of QDs in a polymer block [98].

### 3.6. Ligand-Induced Aggregation

**Figure 9:**
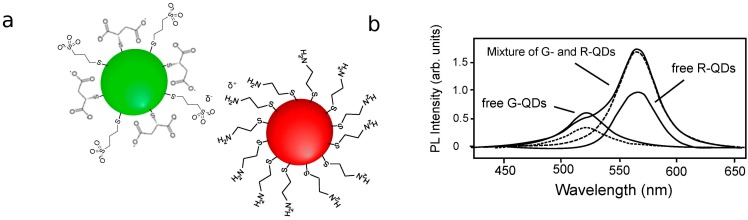
(**a**) Schematic of the QD-QD interactions induced by Wargnier *et al*. QDs coated with a mixture of mecaptosuccinic and mercaptosulfonic acids or cysteamine exhibited negative or positive surface charges, respectively. The opposing charges of these acid and amine terminal groups cause the QDs to aggregate. Schematic is not drawn to scale; (**b**) PL spectra for pure donor (G-QDs), pure acceptor (R-QDs), and mixed dots. The dotted lines represent the contributions from pure donor and acceptors to the mixed population PL; (b) reprinted with permission from [99]. Copyright (2004) American Chemical Society.

FRET studies in colloidal solutions complement those in thin films. Clusters of QDs may be formed by intentionally placing ligands that interact with one another on QD surfaces. Wargnier *et al*. placed oppositely charged ligands on the surface of CdSe/ZnS QDs (Figure 9), inducing aggregation with a range of donor and acceptor concentrations, usually with an excess of donor QDs to increase their molar extinction coefficient [99]. Although significant donor quenching and acceptor PL enhancement was observed in this system, the significant excess of donor QDs unincorporated into a donor-acceptor complex precluded the observation of FRET through time-resolved photoluminescence. This study also raises the question of how to control QD-QD nanoassemblies for efficient device construction. While the authors postulate that the charge-based assembly drives the particles to assemble until the complex reaches a net neutral charge [99], there is little evidence to support whether or not this is how assembly proceeds or to ensure that the smallest possible net neutral clusters are formed rather than larger aggregates.

A more specific version of ligand-induced aggregation involved the high affinity between the protein streptavidin and the vitamin biotin. This interaction is useful for conjugating QDs to each other and to microstructures. For example, Pai *et al*. mixed biotinylated and streptavidin-coated CdSe/ZnS QDs on microspheres of vaterite, a crystalline form of calcium carbonite. The biotin-QD donors and streptavidin-QD acceptors bind to each other to form clusters, turning the vaterite microsphere into a scaffold for streptavidin-biotin conjugated donor-acceptor QD pairs [100].

## 4. QD-QD FRET Biosensors

**Figure 10:**
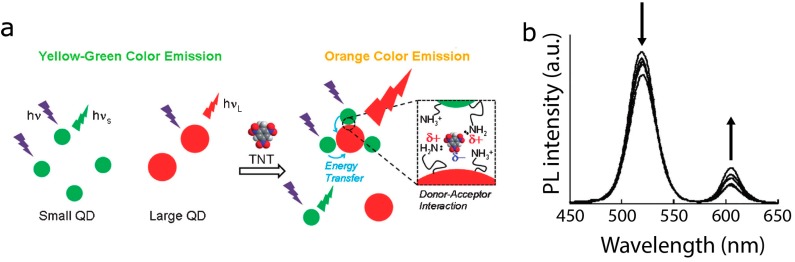
(**a**) Schematic of the TNT sensor by Shiraki *et al*. Positive charges from the amino groups on the QD surface ligands interact with negative charges on the TNT molecule, causing the QDs to aggregate. Schematic is not drawn to scale; (**b**) PL spectra of the sensor with both sizes of QD populations. The arrows indicate how the PL intensity of the donor and acceptors change with increasing analyte concentration. (b) reprinted with permission from [101].

Several biosensors make use of FRET between heterogeneous QDs via analyte-induced aggregation of colloidal QDs. By cleverly selecting the capping ligand of QDs, QDs may specifically aggregate in the presence of a particular chemical or biomolecule. The clustering of QDs causes the PL spectra to shift, indicating the presence of the analyte. For example, the negative charge of the molecule trinitrotoluene (TNT) interacts with the positive charges on terminal amino groups, causing aggregation of QDs capped with amino-terminal PEGs. Shiraki *et al*. used this property to demonstrate a QD-QD FRET-based TNT sensor (Figure 10), reporting a 5 pM detection limit [101]. Because clustering depends on the charge of the TNT molecule, other molecules with similar charge distributions like 2,4-dinitrotoluene and 2-nitrotoluene also induce QD aggregation, albeit less effectively, yielding a lower limit of detection for TNT than for the analogues. This report shows extremely subtle changes in the PL spectra and the time-resolved PL lifetimes for the donor and acceptor QDs, indicating poor FRET efficiencies, but they did quote a lower limit of detection than an alternative competitive assay for TNT based on QD-dye FRET (5 pM *vs*. 88 nM) [102]. It may be that the exceptionally high brightness of both the donor and acceptor moieties in QD-QD FRET enable one to detect relatively fewer binding events, thereby lowering the limit of detection compared to other FRET sensor designs. The antibody-based binding of the QD-dye system, however, clearly holds the advantage in terms of specificity.

**Figure 11:**
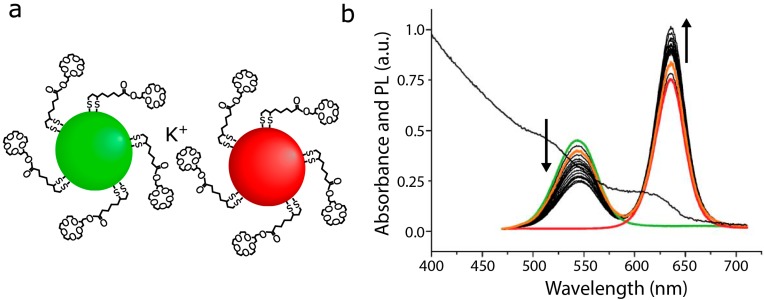
(**a**) Schematic of donor and acceptor QDs coated with 15-crown-5, which selectively interacts with potassium ions. Schematic is not drawn to scale; (**b**) PL spectra of the isolated donor and acceptor QDs (green and red lines, respectively). PL spectra of mixed QD populations after adding 2^n^ × 3.6 μM of KClO_4_ (black lines). Reversibility demonstrated by adding 1 M Na^+^ after the *n*-th addition of K^+^ (orange line). Absorption of spectrum of the QD mixture shown overlaid behind the PL spectra (black line); (b) reprinted with permission from [89].

In a complimentary approach, Ma *et al*. coated two sizes of CdTe QDs in mercaptopropanoic acid (MPA), which generates a negative surface charge. QD clustering and FRET were successfully induced by adding mouse immunoglobulin G (IgG), which has a slight positive charge [103]. However, this sensor is not selective, as any positively charged molecule would presumably induce clustering of QDs in this system. Ion detectors can be made with the same principle. Mayilo *et al*. produced a calcium ion detector with MPA- and thioglycolic acid (TGA)-capped CdTe QDs. The Ca^2+^ ions acted as a chelation linker, inducing QD aggregation (Figure 7) [76]. In principle, other cations, e.g., Mg^2+^ and Zn^2+^, can also act as chelation linkers, so this detector is also not selective. The formation of the QD clusters was reversible through ionic shielding, for example in the presence of sodium carbonate [76]. Chen *et al*. made a K^+^ detector that has a sensitivity limit of 10^−6^ M (Figure 11) [89]. Unlike the Ca^2+^ sensor, the capping ligand 15-crown-5 shows selectivity toward K^+^. The physiological potassium concentration is in the millimolar range [104], making the 15-crown-5 capped QDs potentially useful in biological studies.

Achermann *et al*. used the specific binding of biotin to streptavidin to study microtubule assembly. In this study, QDs were conjugated to streptavidin, while the biotin was conjugated to tubulin monomers. As tubulins polymerized into microtubules, FRET was observed from homotransfer between the QDs in close proximity on the microtubules, serving as confirmation of tubulin assembly. The authors pointed out that energy transfer analysis yields a semi-quantitative understanding of the polymerized microtubule structure as well as the extent of the microtubule assembly [105]. In this unique study, the authors demonstrated that FRET analysis using QDs can be used to study fundamental interactions and dynamics of monomer polymerization. The long lifetime of QDs makes them especially suitable for longitudinal studies such as this.

**Figure 12:**
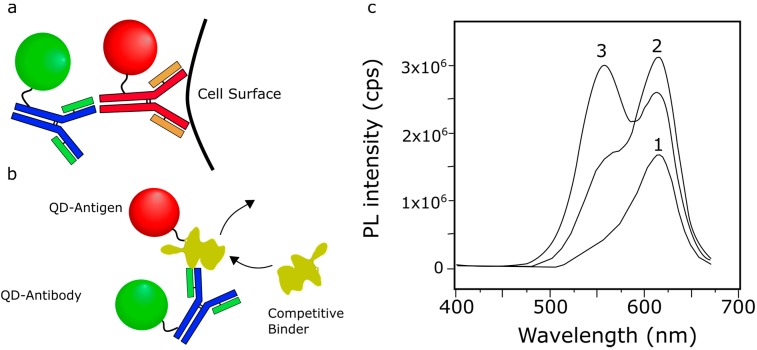
(**a**) Schematic for sensing primary-secondary antibody interaction. Binding of the QD-labeled secondary antibody to the QD-labeled primary antibody brings the donor and acceptor QDs into close proximity, inducing FRET; (**b**) Schematic of competative antibody-antigen binding assay. The QD-labeled antibody binds to a QD-labeled antigen, bringing the donor and acceptor QDs into close enough proximity for efficient energy transfer. In the presence of additional antigen (e.g., an unlabeled endogenous molecule), the QD-labeled antigen is displaced, reducing energy transfer and yielding a measurable change in the PL of the system. Using an unlabeled antigen as the competitive binder reveals the antigen-antibody binding affinity. Schematics are not drawn to scale; (**c**) PL spectra demonstrating that competitive binding can reverse FRET signal. (**c1**) PL spectrum of QD-antibody (acceptors only); (**c2**) PL spectrum of QD-antigen bound to QD-antibodies, showing increased acceptor intensity due to FRET; (**c3**) PL spectrum of the QD-antigen + QD-antibody complex in the presence of the competitive binder, showing an increase in the donor peak intensity and decrease in the acceptor peak intensity; (**c**) Reprinted with permission from [88]. Copyright (2002) American Chemical Society.

In contrast to the non-specific charge-based IgG aggregation described above [103], the specific binding of IgG as a secondary antibody was utilized by Liu *et al*. to induce an interaction between donor and acceptor QDs. HeLa cells served as an anchor for anti-human CD71 monoclonal antibodies, conjugated with donor QDs. They showed that FRET was successfully induced after adding goat anti-mouse IgG-conjugated QDs (Figure 12a) [62]. This shows the successful interaction between anti-CD71 and IgG.

Wang *et al*. developed a biosensor by conjugating CdTe QDs to BSA antigen and anti-BSA (IgG) antibodies (Figure 12b). The formation of BSA-IgG immunocomplex induced FRET in the system, as indicated by the characteristic changes in PL spectra [88]. By using unlabeled BSA in a competitive binding assay, the FRET signal was reversed (Figure 12c), yielding a BSA detector with a detection limit of 10^−8^ M. Similarly, Li *et al*. also used an antibody-antigen interaction (rabbit IgG + anti-rabbit IgG) to induce FRET in a heterogeneous population of CdTe QDs [106]. Their competitive binding assay also reversed the effect of FRET; however, no detection limit was reported [106]. In addition to FRET, both studies confirmed the immunoreaction by using nonspecific and competitive binding assays [88,106]. In this type of interaction, FRET can be used in an assay to detect the presence of an antigen or to measure the antibody-antigen binding affinity. Indeed, Wang *et al*., constructed a detector for *Salmonella enteritidis* detector using anti-*S. enteritidis* antibodies and a secondary antibody to anti-*S. enteritidis* [107]. Both antibodies are conjugated to QDs while the analyte, *S. enteritidis*, acted as the competitive binder to the secondary antibody. The authors reported a limit of detection of 10 CFU/mL. Again in this case the brightness of the QDs overcame the inherently high background of the QD-QD FRET construct to yield a low limit of detection.

In contrast to the primarily solution-phase QD-QD FRET biosensors described above, Seker *et al*. generated film-based QD-QD FRET biosensor using peptide-mediated layer-by-layer assembly of green- and red-emitting donor and acceptor CdTe QDs [108]. By utilizing protease-cleavable polyelectrolyte peptides to cement the QD film structure, the authors concertedly generated films susceptible to enzymatic cleavage, yielding a change in the FRET signal in response to the biochemical reaction. In this paper, the FRET-based analysis was oddly focused primarily on the fluorescence lifetime of the acceptor emission (rather than the fluorescence lifetime of the donor), demonstrating that the lifetime was extended significantly in the FRET-active structured films compared to acceptor-only films [108]. Following protease degradation of the polypeptide structural elements, the film became disordered, reducing the optimization of the energy transfer pathway and shortening the acceptor lifetime. The donors and acceptors did not completely dissociate, as they do in solution-phase, hybrid QD-FRET protease activity assays [109,110], so the effect of the enzymatic degradation was not as pronounced. Although this is a less successful sensor than the well-established alternative protease activity assays, the unique film-based structure of the FRET device is worth noting.

## 5. QD-QD FRET within Complex Nanostructures

There are several advantages to inducing FRET in QDs arranged in complex nanostructures, including: (1) increased FRET efficiency; (2) directed energy transfer; and (3) increased overall absorption cross-section via light harvesting antenna. These desirable properties have been demonstrated in both biosensors and photovoltaic applications.

### 5.1. Increasing FRET Efficiency

**Figure 13:**
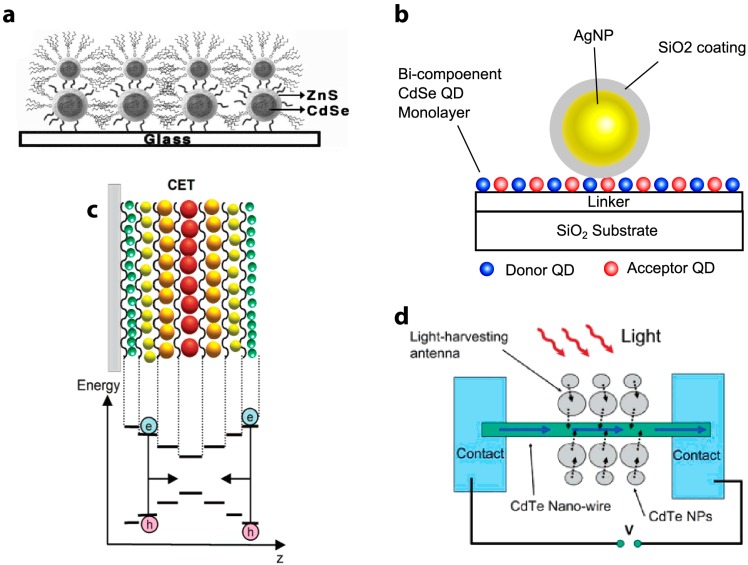
Schematics of QD-QD FRET nanostructures. (**a**) A bilayer structure made by affixing CdSe/ZnS QDs to a glass substrate. Reprinted with permission from [56]; (**b**) A bi-component CdSe QD monolayer with a silver nanoparticle (AgNP) deposited on its surface enables plasmon-enhanced FRET. Reprinted with permission from [116]; (**c**) Schematic of electron funneling along a bandgap gradient. Reprinted with permission from [63]. Copyright (2004) American Chemical Society; (**d**) Schematic example of a photovoltaic made using the principle of bandgap funneling. Reprinted with permission from [111]. Copyright (2005) American Chemical Society. Schematics are not drawn to scale.

One way to guarantee maximal spectral overlap is to align donor and acceptor QDs, creating nanostructures by affixing QDs to a surface or onto each other, such as in a chain [12,105,111] or in layered structures [61,63,112,113,114,115]. Since the overlap integral *J* is optimized by ensuring a donor is always paired with the most suitable acceptor, the maximum theoretical FRET efficiency is higher in such structures. In a bilayered CdSe/ZnS structure, Crooker and coworkers reported a fast donor decay time of τ*_T_* = 750 ps (Figure 13a). In contrast, the donor decay time of CdSe/ZnS QDs in drop-cast thin films is τ*_T_* ~ 20 ns [56]. Recall that the energy transfer rate, *k_T_*, is simply (τ*_T_*)^−1^ (Equation (6)), and shorter times indicate faster energy transfer. The authors commented that this effect is achieved from maximizing the spectral overlap *J* in the donor-acceptor pairs, despite having a smaller number of possible neighboring acceptor molecules.

Another way to improve the rate of energy transfer is to minimize the donor-acceptor distance *R* in the layered structure. Due to the large organic capping ligands, the donor-acceptor distance in the structure reported by Crooker *et al*. was approximately 62 Å [56]. By using oppositely charged capping molecules (TGA and 2-mercaptoethylamine), Franzl *et al*. were able to fabricate CdTe bilayers with minimal space between the QD layers [83]. The absence of TOPO capping ligands and polymer linkers reduced *R* by at least 20 Å. The close proximity of each layer resulted in a fast ET rate of (50 ps)^−1^ [83]. Achermann *et al.* published similar results in CdSe bilayers [112]. With the right configuration, it is predicted that even faster ET rate of (38 ps)^−1^ can achieved [56], but (50 ps)^−1^ is the fastest energy transfer rate reported to date.

Yet another method to improve the energy transfer rate is with surface plasmon resonance, where the dipole in the fluorophore couples with the oscillating surface charges on a metal surface (usually gold or silver nanoparticles) [117]. Govorov *et al*. first described the theory of plasmon-enhanced FRET in semiconductor NCs, although plasmon-enhanced FRET with organic fluorophores had been discussed previously [117,118,119]. Su and coworkers demonstrated that under the right conditions, plasmon coupling increases *R*_0_ and the resulting FRET efficiency [116]. By placing a Ag/SiO_2_ nanoparticle adjacent to a CdSe/ZnS QD monolayer (Figure 13b), the acceptor PL intensity was enhanced 47-fold, and the FRET rate improved from (15 ns)^−1^ to (0.4 ns)^−1^ [116]. Similarly, Lunz *et al*. fabricated a CdTe QD-Au NP-CdTe QD sandwich structure and observed that surface plasmon-enhanced FRET significantly improved the transfer efficiency and increased the working donor-acceptor distance. They reported a FRET rate of (6.5 ns)^−1^ in their sandwich structure [120]. It has also been suggested that it is possible to tune *R*_0_ by adjusting the concentration of the metal nanoparticles, which may be useful for long-distance sensing applications [121]. The inclusion of plasmon coupling also enhanced FRET efficiency and the acceptor emission in a case of interspecies QD-QD FRET, when a Type I CdSe/ZnS QD donated energy to a low QY Type II CdSe/ZnTe acceptor. This was particularly helpful in this case because the low QY (0.01) of the acceptor QD resulted in minimal acceptor emission in the absence of the enhancement [122]. The enhancement is evident specifically when the FRET excitation wavelength corresponds with the plasmon resonance-based absorption feature of the added gold NP [122]. Care must be taken when attempting to use gold and silver particles to enhance energy transfer and the effectiveness of QD-QD FRET devices, because metal NPs also act as non-fluorescent FRET quenchers for QDs [99]. Whether the plasmonic NP acts as a FRET enhancer or a quencher depends on the spectral position of the plasmon resonance peak of the metal NP relative to the absorption and emission spectra of the fluorophores, as well as the distance between the plasmonic structures and the fluorophore.

### 5.2. Directing Energy Transfer

Arranging QDs in nanostructures enables one to direct energy transfer, funneling the charge carrier along a bandgap gradient [56]. Bandgap funnels have been successfully fabricated with 5 layers of different-sized QDs (Figure 13c) [61,63]. The final receiver of the charge carrier can be infrared emitters [61] or current generators for photovoltaic applications (Figure 13d). More recently, it has been shown that in nanocrystal multilayer films, trap-state charge carriers, which usually recombine non-radiatively, can be resonantly transferred to radiative states in nearby large nanocrystals [63,83]. This effectively “recycles” the charge carrier enabling them to once again go through radiative recombination, thus boosting the overall quantum yield of the nanostructure [61,63]. Lee *et al*. reported a four-fold increase in PL when QDs are used to funnel energy carriers to a CdTe nanowire, and Zheng *et al*. showed this configuration has an energy transfer lifetime of 5 ns [12,111]. Similarly, Xu *et al*. reported that the emission of final acceptor in a five-layer funnel improves nineteen-fold compared to a single monolayer [61]. The increased emission is also due to an increased overall energy absorption cross-section, also known as the light harvesting antenna effect [61,112]. Together, these studies suggest energy tunneling across closely packed QD layers is fast and efficient. Increased absorption cross-sections and highly efficient energy transfer results in the possibility of more efficient thin film optoelectronic devices and artificial photosynthetic systems [61,62,83].

### 5.3. Biosensing Using Complex Nanostructures

**Figure 14:**
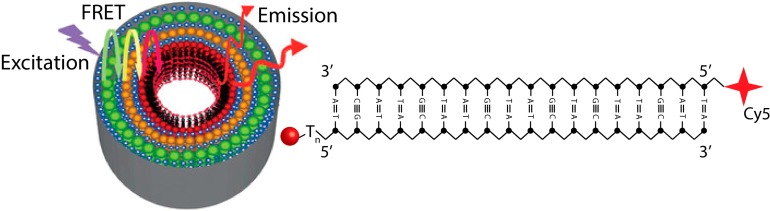
Schematic of the layered QD-nanotube DNA hybridization detection probe. Reprinted with permission from [123]. Schematic is not drawn to scale.

One of the most important benefits of using QDs in biosensing is their high overall brightness compared to organic fluorophores, which comes in large part due to their extremely high molecular extinction coefficients. For this reason, using QDs instead of dyes or FPs in assays can enable the detection of the analyte at a lower concentration. A nanostructure for energy transfer cascade has the benefit of both the light harvesting antenna effect and highly efficient energy transfer, and can thus produce even higher PL intensity than colloidal or thin film QD detectors [61,111]. For example, Feng *et al*. used a layer-by-layer deposition technique to line nanotubes with ZnCdSe QDs, resulting in a graded bandgap structure with three-layers of QDs in a ring structure (Figure 14) [123]. They reported a nine-fold acceptor emission intensity increase [123]. By placing the smaller donor QDs in the outermost rings, the number of donor QDs is maximized, increasing the donor absorption. It has been theorized that in a three-layer QD system, the FRET efficiency could reach 80% [124]. Feng *et al*. reported that with sufficient spectral overlap FRET can induce higher PL from the acceptor QD than direct excitation of the same QDs. The final acceptor in their structure is conjugated to a DNA hybridization detection probe with the organic dye Cy5 as the final energy acceptor. They reported a DNA detection limit of 100 fM, making the QD-lined nanotube a sensitive, highly selective biosensor (Figure 14) [123].

## 6. Discussion

QD-QD FRET sensors vary from the more common QD-dye and QD-FP FRET-based sensors in a couple of significant ways. First, both donor and acceptor moieties in QD-QD systems have multiple binding sites, facilitating binding-induced clustering, which may be used advantageously, but may also be difficult to control. Second, the high molar extinction coefficient in the acceptor QD results in large excitation crosstalk. The bandgap engineering of multicomponent QD-QD FRET devices through either multi-species hetero-FRET or by employing QDs with complex heterostructures has significant potential to improve QD-QD FRET-based sensing by increasing signal-to-noise through the reduction of cross-talk-based background signals while maintaining the high sensitivity of the devices because of the high photon output of the system.

### 6.1. Multivalency in QD-QD FRET Systems

**Figure 15:**
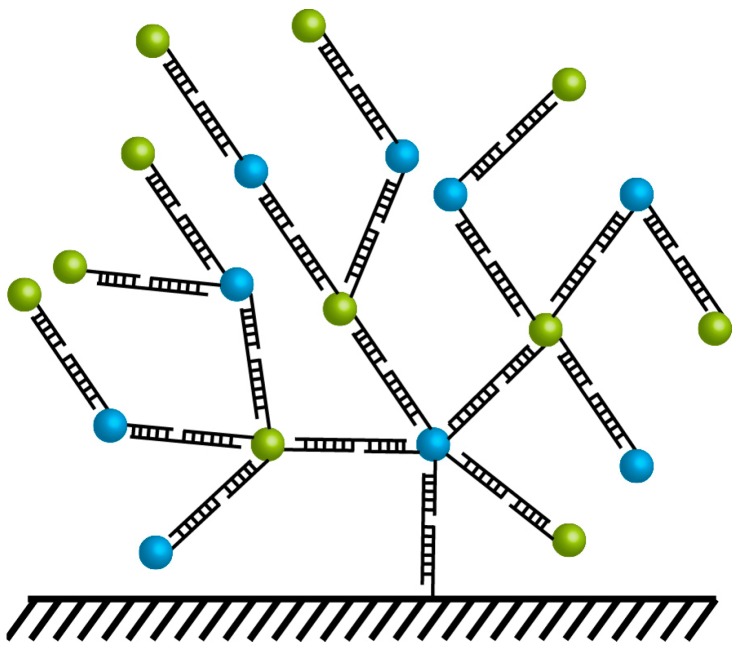
Schematic of surface-tethered induced aggregation, where the presence of a target nucleotide sequence would induce binding of a QD to a surface. The surface-tethered QD would have multiple additional binding sites available for further target binding and additional QDs labeled with complimentary sequences. Such a design is proposed as a means with which to take advantage of the multivalancy of both the donor and acceptor in QD-QD FRET. Schematic is not drawn to scale.

QDs present a large biochemically-active surface, resulting in multiple binding sites. This enables QDs to be successfully utilized as nanoscaffolds in hybrid systems, where multiple organic acceptors bind to a single QD donor, enhancing FRET efficiency (Equation (2), **n** > 1). In addition, the multivalency of the NP has enabled sensor designers to attach multiple labels, delivery sequences, or binding sequences to a single particle, providing for the development of complex multifunctional devices, all centered around one discrete hub. In the case of QD-QD FRET sensors, however, the presence of multiple binding sites on both the donor and acceptor QDs can facilitate significant aggregation, when the donor binds an acceptor, which binds or binds one or more donors, which bind one or more additional acceptors, etc. This form of clustering could be unpredictable and inhomogeneous, which may reduce the repeatability of assays built upon this platform. None of the sensors discussed in this review addressed this aggregation issue, so it is unclear to what extent this impacts sensor design or the reliability of QD-QD FRET-based assays. Solution-phase experiments could certainly be challenged if aggregation were induced to such an extent that colloidal stability was threatened. One could imagine, however, possible sensor designs where induced aggregation could be a significant advantage, for example by raising the local concentration of particles tethered to a surface due to ligand-induced clustering (Figure 15). A surface-tethered sensor could be highly useful for paper-based diagnostics, microfluidic sensors, or TIRF-based imaging, where the analyte-induced aggregation of donor and acceptor QDs could be used to both concentrate the analyte and enhance the detection signal and lower the detection limit.

**Figure 16:**
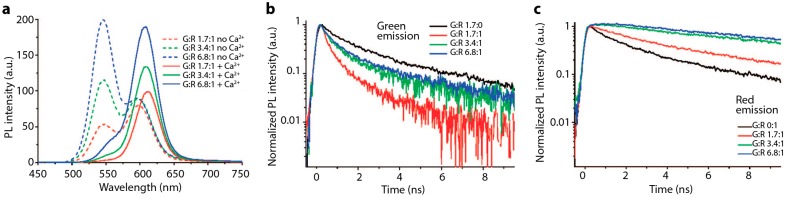
Impact of excess donor on spectral results and time-resolved PL. (**a**) Steady-state PL spectra of mixed green and red, donor and acceptor CdTe QDs with three different donor: acceptor ratios. Spectra shown are for QDs mixed in the absence of Ca^2+^ and QDs mixed in the presence of Ca^2+^, which caused the QDs to associate with each other in close enough proximity to induce FRET; (**b**,**c**) Time-resolved PL decay curved of donor green (**b**) and acceptor red (**c**) QDs in pure green or red samples and in mixed samples in the presence of Ca^2+^. Reprinted with permission from [76]. Copyright (2008) American Chemical Society.

In addition to the various physical donor-acceptor orientations that are possible because of the multivalency of both the donor and acceptor in QD-QD devices, we must also consider the photophysical implications of this structure. In QD-FP or QD-organic dye FRET devices, the most successful sensor designs display multiple acceptors per QD donor. These devices yield increasing FRET efficiencies with each additional acceptor, plateauing once ~4–6 acceptor fluorophores have been successfully attached to the donor QD [22,54]. In contrast, the published QD-QD FRET systems described here often utilize an excess of donor QDs, presumably to increase the amount of the excitation light absorbed by the donor QDs. One can see in an example in Figure 16a that in the absence of FRET (no Ca^2+^, dashed lines) the extra donor QDs increase the donor emission peak intensity with nominal impact on the acceptor peak intensity. In the presence of Ca^2+^, however, the red and green QDs aggregate, bringing them in close enough proximity for FRET. In that case, the excess donor QDs do indeed facilitate increased enhancement of the acceptor emission. When the donor time-resolved PL is examined, one can see that the donor lifetime is shortest with the least excess of donor QDs (Figure 16b). This is to say that the excess of donor QDs actually decreases the FRET efficiency, consistent with Equation (2) and Figure 3. The decrease in efficiency is offset by the improvement in the overall PL signal as more incident light is absorbed by donor dots, resulting in higher total acceptor PL. One can also see in Figure 16c that the acceptor lifetime lengthens with the increase in the number of donor QDs per acceptor [76]. It seems that although less of the total energy absorbed by the donors is transferred to acceptors (Figure 16b), more of the acceptor excitation originates through energy transfer rather than through direct acceptor excitation (Figure 16c). In other words, although the FRET efficiency goes down with the increase in the number of donors per acceptor, the total amount of energy transferred appears to increase. The increase in donor concentration also likely also promotes an increase in donor-to-donor homotransfer, perhaps before or in lieu of energy transfer to the intended acceptor. The QD-QD FRET field is ripe for systematic investigations into the interplay between the donor and acceptor absorption cross-sections, the donor-acceptor ratio, the FRET efficiency, and the ratio between the acceptor and donor emission intensities in response to an analyte. While the studies to date have shown that there is merit to the concept of QD-QD FRET-based sensing, thorough investigation is needed to facilitate concerted optimization of QD-QD FRET sensor design, including optimization of the donor:acceptor ratio.

### 6.2. Crosstalk in QD-QD FRET

The most significant limitation of QD-QD FRET arises as a consequence of high background related to direct acceptor excitation. While we command significant control over the emission properties of the NCs, QD absorption properties are not similarly tailored. Using conventional QDs, significant spectral overlap exists within a donor species, leading to considerable homotransfer. Similarly, high excitation crosstalk leads to significant direct acceptor excitation. Advanced QD compositions may hold promise for addressing these problems. For example, undesired QD homotransfer has been an unfortunate source of self-quenching in QD films intended for solid-state lighting applications. This has been addressed and homotransfer largely eliminated using thick-shelled QDs—So called “giant” nanocrystal quantum dots (g-NQDs) [125]. After many successive shell depositions, the g-NQDs lose their characteristic 1S absorption peak [126]. In theory, the smaller 1S peak in these particles decreases the spectral overlap integral between a donor and itself, resulting in reduced homotransfer. However, it is unclear whether the close-packed films with thicker-shelled QDs exhibit less energy transfer solely because of the larger interparticle distance due to the thicker shell or if there is also a change in the spectral overlap of the particles. The change in optical properties with the change in the composition of the core/shell heterostructure holds potential for tailoring the absorbance spectra of more advanced QD architectures, potentially minimizing the excitation crosstalk by lowering the acceptor absorption at the excitation wavelength. If this can be achieved, it would not be necessary to have excess donors in QD-QD FRET systems, and the transfer efficiency could be enhanced.

### 6.3. Point-Dipole Approximation

As QD energy transfer schemes become more complex and utilize higher order QD heterostructures, additional work is necessary to ensure that the calculations performed to determine the spectral overlap and Förster distance are as accurate as possible. In particular, the assumption that the dipole-dipole interaction is best modeled with a point-dipole approximation, using the center of the QD as the point source (and the QD-QD center-to-center distance as the donor-acceptor distance), fails to account for the true spatial distribution of excitons within a NC. Recent papers show that this assumption is not valid for FRET pairs when the interparticle distance is very small [124] or in complex nanostructures, such as nanotubes [127]. The point-dipole approximation is likely to be challenged further by core/shell heterostructures, particularly non-Type-I core/shells, where the electron and hole are not confined to the nanocrystal core. Further research, both theoretical and experimental, is necessary to demonstrate the appropriate location and orientation of the dipole in complex heterostructured nanoparticles. This is of particular importance with the aforementioned g-NQDs, as the thick shell adds distance between the center of the donor NC and the nearest possible location of the acceptor. If the point-dipole approximation is accurate, then this added distance would reduce FRET efficiency; if the point-dipole approximation misrepresents the true distribution of the dipole within the nanoparticle structure, then our assumptions about the donor-acceptor distance in a thick-shelled heterostructure will prove false.

### 6.4. Future Directions

The QD-QD FRET considerations mentioned thus far, including homotransfer, crosstalk, and multivalency need to be addressed before ideal QD-QD FRET sensors can be realized. The key to addressing these issues lies in the absorption spectra of the quantum dots. As mentioned, a larger Stokes shift can mitigate the effect of homotransfer; using g-NQDs with suppressed 1S peaks could further reduce QD-QD FRET within a nominally monochromatic QD population. It may be possible to address crosstalk in the same way: if donor absorbance is maximized and/or acceptor absorbance is minimized at the excitation wavelength, then excitation crosstalk can be mitigated. Preferential donor excitation would address the issue of low FRET efficiency due to multivalency (**n** < 1) by enabling sufficient signal from fewer donors. The recent advancement of complex heterostructures, such as g-NQDs as well as doped and alloyed QDs, opens the possibility of absorbance spectrum engineering. Using complex-structured QDs for QD-QD FRET may be the key to realizing the ideal QD-QD FRET sensor design.

## 7. Conclusions

The foundational and theoretical work discussed in this review lays the groundwork for the development of sensors using FRET between quantum dot donors and acceptors. FRET sensors utilizing both quantum dot donors and acceptors are attractive due to their presumed photostability compared to quantum dot-organic fluorophore FRET systems, making them promising for longitudinal studies. Their stability makes them good candidates for device-on-a-chip applications and for sensors designed for use outside of the laboratory setting. Their extreme brightness compared to organic fluorophores, further enhanced with energy cascades and light harvesting antenna, enables a lower limit of detection in sensing applications. This yields sensors that are more sensitive than similar sensors made from hybrid systems. However, this field is still underdeveloped and many challenges need to be addressed before such ideal sensors can be fully realized.

Optimal QD-QD FRET devices would utilize donor QDs with: (1) a large molar extinction coefficient at the excitation wavelength; (2) a relatively long fluorescent lifetime compared to the donor moiety; and (3) a high quantum yield. In contrast, the acceptor NC would ideally exhibit (1) a significantly smaller molar extinction coefficient at the excitation wavelength compared to the donor; (2) a shorter fluorescent lifetime than the donor; (3) considerable spectral overlap with the donor, and (4) a high molar extinction coefficient at the donor emission wavelength. In order to achieve donor-acceptor pairs of this description, the donor and acceptor NCs will have to be individually engineered to meet these criteria through interspecies QD-QD FRET, as QDs of the same core composition by definition cannot meet these design specifications. In addition to tuning the photophysics of the donor and acceptor components, the overall sensor design requires more attention to issues of sensor specificity and the impact of aggregation on device reliability. While there is great promise in the field, much concerted and systematic research is necessary to demonstrate that QD-QD FRET-based biosensors can add functionality and utility over better-developed alternatives. The potential for more sensitive and more photostable devices warrants investment into this branch of research.

## References

[1] Förster T. (1948). Zwischenmolekulare Energiewanderung und Fluoreszenz. Ann. Phys..

[2] Lakowicz J.R. (2006). Principles of Fluorescence Spectroscopy.

[3] Zhang Y., Wang T.H. (2012). Quantum dot enabled molecular sensing and diagnostics. Theranostics.

[4] Medintz I.L., Mattoussi H. (2009). Quantum dot-based resonance energy transfer and its growing application in biology. Phys. Chem. Chem. Phys..

[5] Medintz I.L., Uyeda H.T., Goldman E.R., Mattoussi H. (2005). Quantum dot bioconjugates for imaging, labelling and sensing. Nat. Mater..

[6] Yuan L., Lin W., Zheng K., Zhu S. (2013). FRET-based small-molecule fluorescent probes: Rational design and bioimaging applications. Acc. Chem. Res..

[7] Miyawaki A. (2003). Visualization of the spatial and temporal dynamics of intracellular signaling. Dev. Cell.

[8] Claussen J.C., Algar W.R., Hildebrandt N., Susumu K., Ancona M.G., Medintz I.L. (2013). Biophotonic logic devices based on quantum dots and temporally-staggered Förster energy transfer relays. Nanoscale.

[9] Claussen J.C., Hildebrandt N., Susumu K., Ancona M.G., Medintz I.L. (2014). Complex logic functions implemented with quantum dot bionanophotonic circuits. ACS Appl. Mater. Interfaces.

[10] Choi S., Jin H., Bang J., Kim S. (2012). Layer-by-layer quantum dot assemblies for the enhanced energy transfers and their applications toward efficient solar cells. J. Phys. Chem. Lett..

[11] Santra P.K., Kamat P.V. (2013). Tandem-layered quantum dot solar cells: Tuning the photovoltaic response with luminescent ternary cadmium chalcogenides. J. Am. Chem. Soc..

[12] Zheng K., Žídek K., Abdellah M., Torbjörnsson M., Chábera P., Shao S., Zhang F., Pullerits T. (2013). Fast monolayer adsorption and slow energy transfer in CdSe quantum dot sensitized ZnO nanowires. J. Phys. Chem. A.

[13] Kamat P.V. (2008). Quantum dot solar cells. Semiconductor nanocrystals as light harvesters. J. Phys. Chem. C.

[14] Sapsford K.E., Berti L., Medintz I.L. (2006). Materials for fluorescence resonance energy transfer analysis: Beyond traditional donor-acceptor combinations. Angew. Chem. Int. Ed..

[15] Resch-Genger U., Grabolle M., Cavaliere-Jaricot S., Nitschke R., Nann T. (2008). Quantum dots versus organic dyes as fluorescent labels. Nat. Methods.

[16] Alivisatos A.P., Gu W., Larabell C. (2005). Quantum dots as cellular probes. Annu. Rev. Biomed. Eng..

[17] Michalet X., Pinaud F., Lacoste T.D., Dahan M., Bruchez M.P., Alivisatos A.P., Weiss S. (2001). Properties of fluorescent semiconductor nanocrystals and their application to biological labeling. Single Mol..

[18] Alivisatos P. (2004). The use of nanocrystals in biological detection. Nat. Biotechnol..

[19] Dabbousi B.O., Rodriguez-Viejo J., Mikulec F.V., Heine J.R., Mattoussi H., Ober R., Jensen K.F., Bawendi M.G. (1997). (CdSe)ZnS core−shell quantum dots: Synthesis and characterization of a size series of highly luminescent nanocrystallites. J. Phys. Chem. B.

[20] Han M., Gao X., Su J.Z., Nie S. (2001). Quantum-dot-tagged microbeads for multiplexed optical coding of biomolecules. Nat. Biotechnol..

[21] Klostranec J.M., Chan W.C.W. (2006). Quantum dots in biological and biomedical research: Recent progress and present challenges. Adv. Mater..

[22] Clapp A.R., Medintz I.L., Mauro J.M., Fisher B.R., Bawendi M.G., Mattoussi H. (2004). Fluorescence resonance energy transfer between quantum dot donors and dye-labeled protein acceptors. J. Am. Chem. Soc..

[23] Clapp A.R., Medintz I.L., Mattoussi H. (2006). Förster resonance energy transfer investigations using quantum-dot fluorophores. Chemphyschem.

[24] Sapsford K.E., Pons T., Medintz I.L., Mattoussi H. (2006). Biosensing with luminescent semiconductor quantum dots. Sensors.

[25] Chen G.W., Song F.L., Xiong X.Q., Peng X.J. (2013). Fluorescent nanosensors based on fluorescence resonance energy transfer (FRET). Ind. Eng. Chem. Res..

[26] Chen N.T., Cheng S.H., Liu C.P., Souris J.S., Chen C.T., Mou C.Y., Lo L.W. (2012). Recent advances in nanoparticle-based Förster resonance energy transfer for biosensing, molecular imaging and drug release profiling. Int. J. Mol. Sci..

[27] Petryayeva E., Algar W.R., Medintz I.L. (2013). Quantum dots in bioanalysis: A review of applications across various platforms for fluorescence spectroscopy and imaging. Appl. Spectrosc..

[28] Clapp A.R., Medintz I.L., Fisher B.R., Anderson G.P., Mattoussi H. (2005). Can luminescent quantum dots be efficient energy acceptors with organic dye donors?. J. Am. Chem. Soc..

[29] Algar W.R., Wegner D., Huston A.L., Blanco-Canosa J.B., Stewart M.H., Armstrong A., Dawson P.E., Hildebrandt N., Medintz I.L. (2012). Quantum dots as simultaneous acceptors and donors in time-gated Förster resonance energy transfer relays: Characterization and biosensing. J. Am. Chem. Soc..

[30] Geissler D., Linden S., Liermann K., Wegner K.D., Charbonniere L.J., Hildebrandt N. (2014). Lanthanides and quantum dots as Förster resonance energy transfer agents for diagnostics and cellular imaging. Inorg. Chem..

[31] So M.-K., Loening A.M., Gambhir S.S., Rao J. (2006). Creating self-illuminating quantum dot conjugates. Nat. Protoc..

[32] So M.K., Xu C.J., Loening A.M., Gambhir S.S., Rao J.H. (2006). Self-illuminating quantum dot conjugates for *in vivo* imaging. Nat. Biotechnol..

[33] Yao H.Q., Zhang Y., Xiao F., Xia Z.Y., Rao J.H. (2007). Quantum dot/bioluminescence resonance energy transfer based highly sensitive detection of proteases. Angew. Chem. Int. Ed..

[34] Huang X.Y., Li L., Qian H.F., Dong C.Q., Ren J.C. (2006). A resonance energy transfer between chemiluminescent donors and luminescent quantum-dots as acceptors (CRET). Angew. Chem..

[35] Chan W.C.W., Maxwell D.J., Gao X.H., Bailey R.E., Han M.Y., Nie S.M. (2002). Luminescent quantum dots for multiplexed biological detection and imaging. Curr. Opin. Biotechnol..

[36] Baskoutas S., Terzis A.F. (2006). Size-dependent band gap of colloidal quantum dots. J. Appl. Phys..

[37] Hines M.A., Guyot-Sionnest P. (1996). Synthesis and characterization of strongly luminescing ZnS-capped CdSe nanocrystals. J. Phys. Chem..

[38] Cox J. (2003). A quantum paintbox. Chem Brit..

[39] Smith A.M., Nie S.M. (2010). Semiconductor nanocrystals: Structure, properties, and band gap engineering. Accounts Chem. Res..

[40] Nam J., Won N., Bang J., Jin H., Park J., Jung S., Jung S., Park Y., Kim S. (2013). Surface engineering of inorganic nanoparticles for imaging and therapy. Adv. Drug Deliv. Rev..

[41] Biju V. (2014). Chemical modifications and bioconjugate reactions of nanomaterials for sensing, imaging, drug delivery and therapy. Chem. Soc. Rev..

[42] Hines D.A., Kamat P.V. (2014). Recent advances in quantum dot surface chemistry. ACS Appl. Mater. Inter..

[43] Tyrakowski C.M., Snee P.T. (2014). A primer on the synthesis, water-solubilization, and functionalization of quantum dots, their use as biological sensing agents, and present status. Phys. Chem. Chem. Phys..

[44] Klimov V.I., McBranch D.W., Leatherdale C.A., Bawendi M.G. (1999). Electron and hole relaxation pathways in semiconductor quantum dots. Phys. Rev. B.

[45] Reiss P., Protière M., Li L. (2009). Core/shell semiconductor nanocrystals. Small.

[46] Lunz M., Bradley A.L., Chen W.Y., Gerard V.A., Byrne S.J., Gun’Ko Y.K., Lesnyak V., Gaponik N. (2010). Influence of quantum dot concentration on Förster resonant energy transfer in monodispersed nanocrystal quantum dot monolayers. Phys. Rev. B.

[47] Dennis A.M., Rhee W.J., Sotto D., Dublin S.N., Bao G. (2012). Quantum dot-fluorescent protein FRET probes for sensing intracellular pH. ACS Nano.

[48] Mićić O.I., Sprague J., Lu Z., Nozik A.J. (1996). Highly efficient band-edge emission from InP quantum dots. Appl. Phys. Lett..

[49] Wu P., Yan X.P. (2013). Doped quantum dots for chemo/biosensing and bioimaging. Chem. Soc. Rev..

[50] Mahler B., Spinicelli P., Buil S., Quelin X., Hermier J.P., Dubertret B. (2008). Towards non-blinking colloidal quantum dots. Nat. Mater..

[51] Chen Y., Vela J., Htoon H., Casson J.L., Werder D.J., Bussian D.A., Klimov V.I., Hollingsworth J.A. (2008). “Giant” multishell CdSe nanocrystal quantum dots with suppressed blinking. J. Am. Chem. Soc..

[52] Van der Meer B.W. (2013). Förster Theory. FRET—Förster Resonance Energy Transfer.

[53] Hildebrandt N. (2013). How to apply FRET: From experimental design to data analysis. FRET—Förster Resonance Energy Transfer.

[54] Dennis A.M., Bao G. (2008). Quantum dot-fluorescent protein pairs as novel fluorescence resonance energy transfer probes. Nano Lett..

[55] Bose R., McMillan J.F., Gao J., Rickey K.M., Chen C.J., Talapin D.V., Murray C.B., Wong C.W. (2008). Temperature-tuning of near-infrared monodisperse quantum dot solids at 1.5 microm for controllable Förster energy transfer. Nano Lett..

[56] Crooker S.A., Hollingsworth J.A., Tretiak S., Klimov V.I. (2002). Spectrally resolved dynamics of energy transfer in quantum-dot assemblies: Towards engineered energy flows in artificial materials. Phys. Rev. Lett..

[57] Muñoz-Losa A., Curutchet C., Krueger B.P., Hartsell L.R., Mennucci B. (2009). Fretting about FRET: Failure of the ideal dipole approximation. Biophys. J..

[58] Fluorophores.org. http://www.fluorophores.tugraz.at/.

[59] Hink M.A., Visser N.V., Borst J.W., van Hoek A., Visser A.J.W.G. (2003). Practical use of corrected fluorescence excitation and emission spectra of fluorescent proteins in Förster resonance energy transfer (FRET) studies. J. Fluoresc..

[60] Olenych S.G., Claxton N.S., Ottenberg G.K., Davidson M.W. (2007). The fluorescent protein color palette. Curr. Protoc. Cell Biol..

[61] Xu F., Ma X., Haughn C.R., Benavides J., Doty M.F., Cloutier S.G., Pbs C., Dot Q. (2011). Efficient exciton funneling in cascaded PbS quantum dot superstructures. ACS Nano.

[62] Liu T.-C., Zhang H.-L., Wang J.-H., Wang H.-Q., Zhang Z.-H., Hua X.-F., Cao Y.-C., Luo Q.-M., Zhao Y.-D. (2008). Study on molecular interactions between proteins on live cell membranes using quantum dot-based fluorescence resonance energy transfer. Anal. Bioanal. Chem..

[63] Franzl T., Klar T.A., Schietinger S., Rogach A.L., Feldmann J. (2004). Exciton recycling in graded gap nanocrystal structures. Nano Lett..

[64] Broussard J.A., Rappaz B., Webb D.J., Brown C.M. (2013). Fluorescence resonance energy transfer microscopy as demonstrated by measuring the activation of the serine/threonine kinase Akt. Nat. Protoc..

[65] Geissler D., Stufler S., Lohmannsroben H.G., Hildebrandt N. (2013). Six-color time-resolved Förster resonance energy transfer for ultrasensitive multiplexed biosensing. J. Am. Chem. Soc..

[66] Luchowski R., Matveeva E.G., Gryczynski I., Terpetschnig E.A., Patsenker L., Laczko G., Borejdo J., Gryczynski Z. (2008). Single molecule studies of multiple-fluorophore labeled antibodies. Effect of homo-FRET on the number of photons available before photobleaching. Curr. Pharm. Biotechnol..

[67] Grundmann M., Christen J., Ledentsov N.N., Böhrer J., Bimberg D., Ruvimov S.S., Werner P., Richter U., Gösele U., Heydenreich J. (1995). Ultranarrow luminescence lines from single quantum dots. Phys. Rev. Lett..

[68] Valerini D., Creti A., Lomascolo M., Manna L., Cingolani R., Anni M. (2005). Temperature dependence of the photoluminescence properties of colloidal CdSe/ZnS core/shell quantum dots embedded in a polystyrene matrix. Phys. Rev. B.

[69] Micic O.I., Jones K.M., Cahill A., Nozik A.J. (1998). Optical, electronic, and structural properties of uncoupled and close-packed arrays of InP quantum dots. J. Phys. Chem. B.

[70] Li J.J., Wang Y.A., Guo W.Z., Keay J.C., Mishima T.D., Johnson M.B., Peng X.G. (2003). Large-scale synthesis of nearly monodisperse CdSe/CdS core/shell nanocrystals using air-stable reagents via successive ion layer adsorption and reaction. J. Am. Chem. Soc..

[71] Murray C.B., Norris D.J., Bawendi M.G. (1993). Synthesis and characterization of nearly monodisperse CdE (E = S, Se, Te) semiconductor nanocrystallites. J. Am. Chem. Soc..

[72] Talapin D.V., Rogach A.L., Kornowski A., Haase M., Weller H. (2001). Highly luminescent monodisperse CdSe and CdSe/ZnS nanocrystals synthesized in a hexadecylamine-trioctylphosphine oxide-trioctylphospine mixture. Nano Lett..

[73] Lingley Z., Lu S., Madhukar A. (2011). A high quantum efficiency preserving approach to ligand exchange on lead sulfide quantum dots and interdot resonant energy transfer. Nano Lett..

[74] Clark S.W., Harbold J.M., Wise F.W. (2007). Resonant energy transfer in PbS quantum dots. J. Phys. Chem. C.

[75] Kagan C.R., Murray C.B., Nirmal M., Bawendi M.G. (1996). Electronic energy transfer in CdSe quantum dot solids. Phys. Rev. Lett..

[76] Mayilo S., Hilhorst J., Susha A.S., Höhl C., Franzi T., Klar T.A., Rogach A.L., Feldmann J. (2008). Energy transfer in solution-based clusters of CdTe nanocrystals electrostatically bound by calcium ions. J. Phys. Chem. C.

[77] Shepherd D.P., Whitcomb K.J., Milligan K.K., Goodwin P.M., Gelfand M.P., van Orden A. (2010). Fluorescence intermittency and energy transfer in small clusters of semiconductor quantum dots. J. Phys. Chem. C.

[78] Tang Z., Ozturk B., Wang Y., Kotov N.A. (2004). Simple preparation strategy and one-dimensional energy transfer in CdTe nanoparticle chains. J. Phys. Chem. B.

[79] Xu L., Xu J., Li W., Zhao W., Sun P., Ma Z., Huang X., Chen K. (2007). Luminescence and resonant energy transfer of two sizes of CdTe quantum dots embedded in gelatin films. J. Mater. Sci..

[80] Thuy U.T.D., Thuy P.T., Liem N.Q., Li L., Reiss P. (2010). Comparative photoluminescence study of close-packed and colloidal InP/ZnS quantum dots. Appl. Phys. Lett..

[81] Kagan C.R., Murray C.B., Bawendi M.G. (1996). Long-range resonance transfer of electronic excitations in close-packed CdSe quantum-dot solids. Phys. Rev. B.

[82] Miyazaki J., Kinoshita S., Jin T. (2011). Non-radiative exciton recombination through excitation energy transfer in quantum dot clusters. J. Lumin..

[83] Franzl T., Shovel A., Rogach A.L., Gaponik N., Klar T.A., Eychmüller A., Feldmann J. (2005). High-rate unidirectional energy transfer in directly assembled CdTe nanocrystal bilayers. Small.

[84] Yatsui T., Jeong H., Ohtsu M. (2008). Controlling the energy transfer between near-field optically coupled ZnO quantum dots. Appl. Phys. B.

[85] Wang C.H., Chen C.W., Wei C.M., Chen Y.F., Lai C.W., Ho M.L., Chou P.T. (2009). Resonant energy transfer between CdSe/ZnS Type I and CdSe/ZnTe Type II quantum dots. J. Phys. Chem. C.

[86] Sarkar S., Maity A.R., Karan N.S., Pradhan N. (2013). Fluorescence energy transfer from doped to undoped quantum dots. J. Phys. Chem C.

[87] Ebenstein Y., Yoskovitz E., Costi R., Aharoni A., Banin U. (2006). Interaction of scanning probes with semiconductor nanocrystals; physical mechanism and basis for near-field optical imaging. J. Phys. Chem. A.

[88] Wang S., Mamedova N., Kotov N.A., Chen W., Studer J. (2002). Antigen/antibody immunocomplex from CdTe nanoparticle bioconjugates. Nano Lett..

[89] Chen C.-Y., Cheng C.-T., Lai C.-W., Wu P.-W., Wu K.-C., Chou P.-T., Chou Y.-H., Chiu H.-T. (2006). Potassium ion recognition by 15-crown-5 functionalized CdSe/ZnS quantum dots in H_2_O. Chem. Commun..

[90] Lü W., Umezu I., Sugimura A. (2008). Evolution of energy transfer process between quantum dots of two different sizes during the evaporation of solvent. Jpn. J. Appl. Phys..

[91] Xu L., Xu J., Ma Z., Li W., Huang X., Chen K. (2006). Direct observation of resonant energy transfer between quantum dots of two different sizes in a single water droplet. Appl. Phys. Lett..

[92] Maenosono S., Dushkin C.D., Saita S., Yamaguchi Y. (1999). Growth of a semiconductor nanoparticle ring during the drying of a suspension droplet. Langmuir.

[93] Yu W.W., Qu L., Guo W., Peng X. (2003). Experimental determination of the extinction coefficient of CdTe, CdSe, and CdS nanocrystals. Chem. Mater..

[94] De Geyter B., Hens Z. (2010). The absorption coefficient of PbSe/CdSe core/shell colloidal quantum dots. Appl. Phys. Lett..

[95] Chen C.W., Wang C.H., Chen Y.F., Lai C.W., Chou P.T. (2008). Tunable energy transfer efficiency based on the composite of mixed CdSe quantum dots and elastomeric film. Appl. Phys. Lett..

[96] Generalova A.N., Oleinikov V.A., Sukhanova A., Artemyev M.V., Zubov V.P., Nabiev I. (2013). Quantum dot-containing polymer particles with thermosensitive fluorescence. Biosens. Bioelectron..

[97] Tomczak N., Janczewski D., Han M.Y., Vancso G.J. (2009). Designer polymer-quantum dot architectures. Prog. Polym. Sci..

[98] Lee J., Sundar V.C., Heine J.R., Bawendi M.G., Jensen K.F. (2000). Full color emission from II-VI semiconductor quantum dot-polymer composites. Adv. Mater..

[99] Wargnier R., Baranov A.V., Maslov V.G., Stsiapura V., Artemyev M., Pluot M., Sukhanova A., Nabiev I. (2004). Energy transfer in aqueous solutions of oppositely charged CdSe/ZnS core/shell quantum dots and in quantum dot−nanogold assemblies. Nano Lett..

[100] Pai R.K., Cotlet M. (2011). Highly stable, water-soluble, intrinsic fluorescent hybrid scaffolds for imaging and biosensing. J. Phys. Chem. C.

[101] Shiraki T., Tsuchiya Y., Shinkai S. (2010). Ratiometric fluorescent sensor for 2,4,6-trinitrotoluene designed based on energy transfer between size-different quantum dots. Chem. Lett..

[102] Goldman E.R., Medintz I.L., Whitley J.L., Hayhurst A., Clapp A.R., Uyeda H.T., Deschamps J.R., Lassman M.E., Mattoussi H. (2005). A hybrid quantum dot-antibody fragment fluorescence resonance energy transfer-based TNT sensor. J. Am. Chem. Soc..

[103] Ma Q., Su X.G., Wang X.Y., Wan Y., Wang C.L., Yang B., Jin Q.H. (2005). Fluorescence resonance energy transfer in doubly-quantum dot labeled IgG system. Talanta.

[104] Boron W.F., Boulpaep E.L. (2005). Medical Physiology: A Cellular and Molecular Approach.

[105] Achermann M., Jeong S., Balet L., Montano G., Hollingsworth J.A. (2011). Efficient quantum dot-quantum dot and quantum dot-dye energy transfer in biotemplated assemblies. ACS Nano.

[106] Li Y., Ma Q., Wang X.Y., Su X.G. (2007). Fluorescence resonance energy transfer between two quantum dots with immunocomplexes of antigen and antibody as a bridge. Luminescence.

[107] Wang B.B., Wang Q., Jin Y.G., Ma M.H., Cai Z.X. (2015). Two-color quantum dots-based fluorescence resonance energy transfer for rapid and sensitive detection of Salmonella on eggshells. J. Photochem. Photobiol. A.

[108] Seker U.O.S., Ozel T., Demir H.V. (2011). Peptide-mediated constructs of quantum dot nanocompositesfor enzymatic control of nonradiative energy transfer. Nano Lett..

[109] Boeneman K., Mei B.C., Dennis A.M., Bao G., Deschamps J.R., Mattoussi H., Medintz I.L. (2009). Sensing caspase 3 activity with quantum dot-fluorescent protein assemblies. J. Am. Chem. Soc..

[110] Medintz I.L., Clapp A.R., Brunel F.M., Tiefenbrunn T., Uyeda H.T., Chang E.L., Deschamps J.R., Dawson P.E., Mattoussi H. (2006). Proteolytic activity monitored by fluorescence resonance energy transfer through quantum-dot-peptide conjugates. Nature Mater..

[111] Lee J., Govorov A.O., Kotov N.A. (2005). Bioconjugated superstructures of CdTe nanowires and nanoparticles: Multistep cascade Förster resonance energy transfer and energy channeling. Nano Lett..

[112] Achermann M., Petruska M.A., Crooker S.A., Klimov V.I. (2003). Picosecond energy transfer in quantum dot Langmuir−Blodgett nanoassemblies. J. Phys. Chem. B.

[113] Lunz M., Bradley A.L., Gerard V.A., Byrne S.J., Gun’Ko Y.K., Lesnyak V., Gaponik N. (2011). Concentration dependence of Förster resonant energy transfer between donor and acceptor nanocrystal quantum dot layers: Effect of donor-donor interactions. Phys. Rev. B.

[114] De Benedetti W.J.I., Nimmo M.T., Rupich S.M., Caillard L.M., Gartstein Y.N., Chabal Y.J., Malko A.V. (2014). Efficient directed energy transfer through size-gradient nanocrystal layers into silicon substrates. Adv. Funct. Mater..

[115] Xu F., Haughn C.R., Ma X., Doty M.F., Cloutier S.G. (2014). Charge-Transfer dynamics in multilayered PbS and PbSe quantum dot architectures. Appl. Phys. Lett..

[116] Su X.-R., Zhang W., Zhou L., Peng X.-N., Wang Q.-Q. (2010). Plasmon-enhanced Förster energy transfer between semiconductor quantum dots: Multipole effects. Opt. Express.

[117] Lakowicz J.R. (2001). Radiative decay engineering: Biophysical and biomedical applications. Anal. Biochem..

[118] Hua X.M., Gersten J.I., Nitzan A. (1985). Theory of energy transfer between molecules near solid state particles. J. Chem. Phys..

[119] Govorov A.O., Lee J., Kotov N.A. (2007). Theory of plasmon-enhanced Förster energy transfer in optically-excited semiconductor and metal nanoparticles. Phys. Rev. B.

[120] Lunz M., Gerard V.A., Gun’ko Y.K., Lesnyak V., Gaponik N., Susha A.S., Rogach A.L., Bradley A.L. (2011). Surface plasmon enhanced energy transfer between donor and acceptor CdTe nanocrystal quantum dot monolayers. Nano Lett..

[121] Zhang X., Marocico C.A., Lunz M., Gerard V.A., Gun’Ko Y.K., Lesnyak V., Gaponik N., Susha A.S., Rogach A.L., Bradley A.L. (2014). Experimental and theoretical investigation of the distance dependence of localized surface plasmon coupled Förster resonance energy transfer. ACS Nano.

[122] Wang C.H., Chen C.W., Chen Y.T., Wei C.M., Chen Y.F., Lai C.W., Ho M.L., Chou P.T., Hofmann M. (2010). Surface plasmon enhanced energy transfer between type I CdSe/ZnS and type II CdSe/ZnTe quantum dots. Appl. Phys. Lett..

[123] Feng C.L., Zhong X.H., Steinhart M., Caminade A.M., Majoral J.P., Knoll W. (2008). Functional quantum-dot/dendrimer nanotubes for sensitive detection of DNA hybridization. Small.

[124] Zheng K., Žídek K., Abdellah M., Zhu N., Chábera P., Lenngren N., Chi Q., Pullerits T. (2014). Directed energy transfer in films of CdSe quantum dots: Beyond the point dipole approximation. J. Am. Chem. Soc..

[125] Kundu J., Ghosh Y., Dennis A.M., Htoon H., Hollingsworth J.A. (2012). Giant nanocrystal quantum dots: stable down-conversion phosphors that exploit a large stokes shift and efficient shell-to-core energy relaxation. Nano Lett..

[126] Pal B.N., Ghosh Y., Brovelli S., Laocharoensuk R., Klimov V.I., Hollingsworth J.A., Htoon H. (2012). ‘Giant’ CdSe/CdS core/shell nanocrystal quantum dots as efficient electroluminescent materials: Strong influence of shell thickness on light-emitting diode performance. Nano Lett..

[127] Wong C.Y., Curutchet C., Tretiak S., Scholes G.D. (2009). Ideal dipole approximation fails to predict electronic coupling and energy transfer between semiconducting single-wall carbon nanotubes. J. Chem. Phys..

